# Transcranial Current Stimulation During Sleep Facilitates Insight into Temporal Rules, but does not Consolidate Memories of Individual Sequential Experiences

**DOI:** 10.1038/s41598-018-36107-7

**Published:** 2019-02-06

**Authors:** Itamar Lerner, Nicholas A. Ketz, Aaron P. Jones, Natalie B. Bryant, Bradley Robert, Steven W. Skorheim, Arno Hartholt, Albert S. Rizzo, Mark A. Gluck, Vincent P. Clark, Praveen K. Pilly

**Affiliations:** 1 Center of Molecular and Behavior Neuroscience, Rutgers University – Newark, 197 University Ave., Newark, NJ 07102 USA; 2 Center for Human-Machine Collaboration, Information and Systems Sciences Laboratory, HRL Laboratories, LLC, Malibu, CA 90265 USA; 3 Psychology Clinical Neuroscience Center, University of New Mexico. Logan Hall, MSC03-2220, 1 University of New Mexico, Albuquerque, NM 87131-0001 USA; 4 Institute for Creative Technologies, University of Southern California, 12015 Waterfront Drive, Playa Vista, CA 90094-2536 USA

## Abstract

Slow-wave sleep (SWS) is known to contribute to memory consolidation, likely through the reactivation of previously encoded waking experiences. Contemporary studies demonstrate that when auditory or olfactory stimulation is administered during memory encoding and then reapplied during SWS, memory consolidation can be enhanced, an effect that is believed to rely on targeted memory reactivation (TMR) induced by the sensory stimulation. Here, we show that transcranial current stimulations (tCS) during sleep can also be used to induce TMR, resulting in the facilitation of high-level cognitive processes. Participants were exposed to repeating sequences in a realistic 3D immersive environment while being stimulated with particular tCS patterns. A subset of these tCS patterns was then reapplied during sleep stages N2 and SWS coupled to slow oscillations in a closed-loop manner. We found that in contrast to our initial hypothesis, performance for the sequences corresponding to the reapplied tCS patterns was no better than for other sequences that received stimulations only during wake or not at all. In contrast, we found that the more stimulations participants received overnight, the more likely they were to detect temporal regularities governing the learned sequences the following morning, with tCS-induced beta power modulations during sleep mediating this effect.

## Introduction

Sleep plays an important role in consolidating recently encoded memories and facilitating a variety of cognitive skills^1^. The consolidation process for declarative memories is thought to involve the coordinated transfer of memory traces from short-term fast-learning storage in the hippocampus to long-term slow-learning storage across the neocortex, occurring mostly during slow-wave sleep (SWS). Evidence from multi-site *in vivo* recordings in rodents during SWS^2,3^ and fMRI recordings in humans during waking periods of rest^4,5^ shows that the fidelity of the consolidation process, as manifested in stronger subsequent recall of the learned stimuli, may be related to the reactivations, or replay, of the spatiotemporal brain activity patterns that were elicited during learning at wake. Specifically, coordinated replay across the hippocampus and neocortex – as indexed by enhanced slow-wave (SW) oscillations^6^, increased spindle-SW phase-amplitude coupling^7,8^, and enhanced functional connectivity between hippocampal and cortical voxels^4,9^, among others – may allow for the consolidation of waking experiences in the neocortex^10^ and result in the facilitation of subsequent memory recall performance during waking^11,12^. Accordingly, over the last decade and a half, researchers have developed non-invasive methods to modulate both memory consolidation during sleep and its manifestations in brain oscillations using either sensory or electric stimulation. In particular, auditory, olfactory and transcranial current stimulation have been shown to boost SW oscillations and/or fast spindles, as well as improve post-sleep declarative memory performance^6,7,13–18^. Among these, Targeted Memory Reactivation (TMR) techniques demonstrated that the consolidation of individual memories could be modulated to some extent during sleep, by first associating specific learning events with auditory or olfactory cues during waking and then reintroducing these cues during SWS, resulting in improved post-sleep performance for these particular memories^13,15,19^.

Nevertheless, investigations of cueing during sleep have also been limited in several aspects. First, studies have focused on the consolidation of simple declarative and procedural memories, and rarely attempted to examine whether cueing can also facilitate a variety of higher-level cognitive processes that are known to be expedited by SWS, such as gaining insight into hidden rules, detecting high-order stimuli relations, and learning of statistical regularities^20–27^. Second, the ability to associate sensory (i.e., auditory or olfactory) cues with individual memories and motor sequences has only been demonstrated in controlled laboratory settings^13,15^. Sensory cues, however, might not be practical for associating with events in the real world where they may need to compete with ambient environmental stimuli. Moreover, the parameters for sensory cueing need to be optimized such that they are sufficiently strong to elicit TMR but not exceedingly strong to awaken the users from their sleep. Finally, the number of discriminable sensory cues that can be associated with real-world memories may not be scalable to the increasing diversity of events that individuals experience over their lifetime.

**Figure 1:**
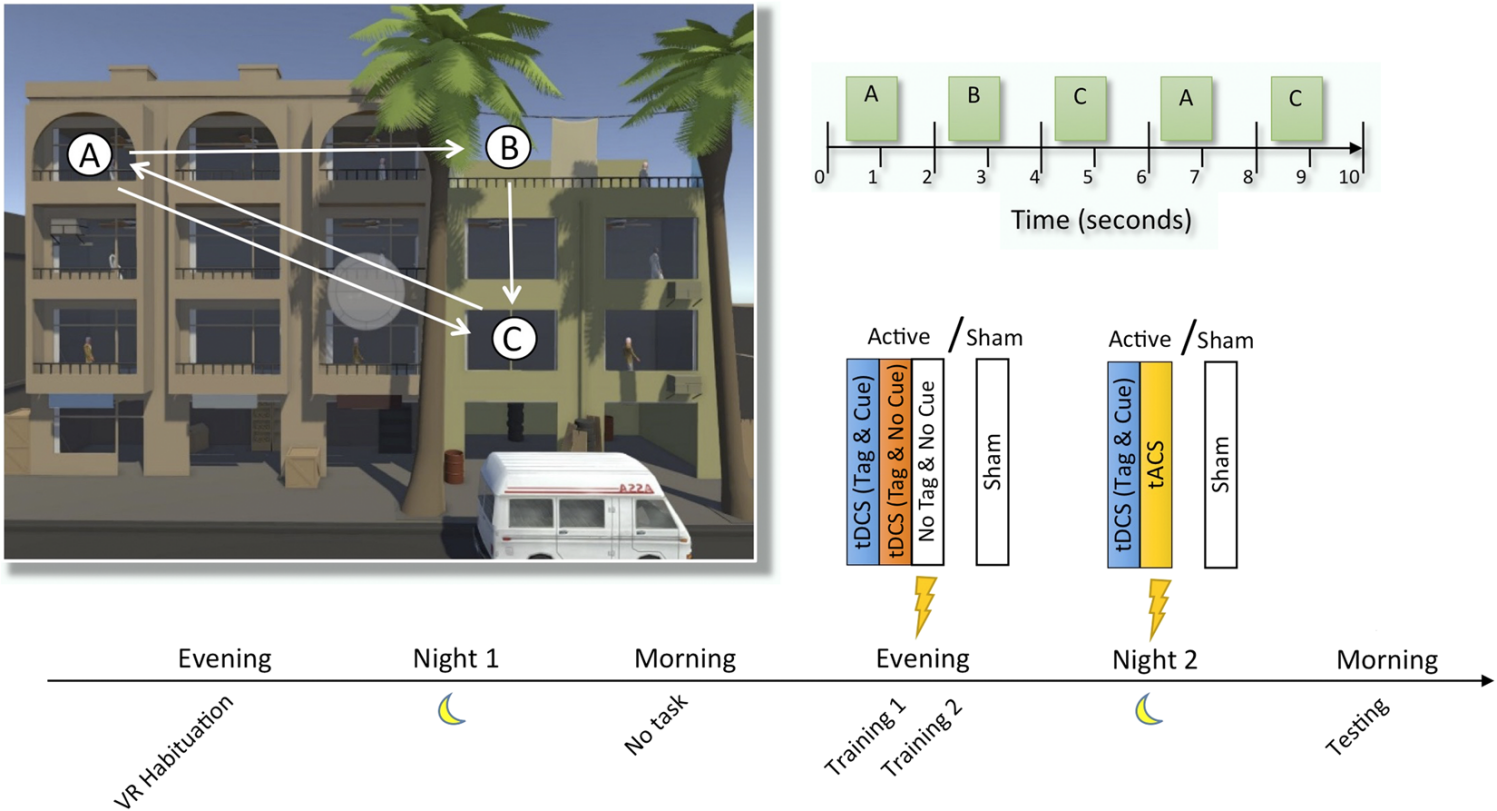
Illustration of the behavioral task. The 3D designed virtual reality (VR) task employed in this study involved six separate sequences of characters (targets) appearing in different windows. Each sequence contained 5 characters popping up one after the other, but following a structure where the first and third characters reappeared in the same windows in positions four and five in the sequence, respectively, forming an A-B-C-A-C order. Subjects’ objective was to aim their sight towards each target and ‘take a picture’ as quickly as possible. The first night in the laboratory was a habituation night before which the subjects were introduced to the VR setup and task. The following evening subjects performed two training sessions during which they received tDCS STAMPs to tag some of the sequences in the task (or no stimulation in the Sham group). They then slept overnight in the lab while receiving additional tDCS STAMPs and tACS (or Sham). Finally, the following morning, they performed a test session. See text for details.

In light of these limitations, in the current study we sought to examine whether application of transcranial current stimulation during sleep can, on the one hand, enhance the recall of individual sequential memories in naturalistic settings within a virtual 3D environment; and, on the other hand, enhance insight into a hidden rule that characterizes the memories as a whole. In the 3D environment, subjects reacted, as quickly as possible, to sequences of 5 targets appearing in succession (Fig. 1; see Materials and Methods for details, and Movie S1 in Supplementary Information for a video demonstration). The hidden rule consisted of temporal regularities in the relative order of the targets across sequences, recently suggested to be the most sensitive to sleep-dependent insight^28^. Subjects’ implicit knowledge of the sequences was measured, upon each target’s onset, as the distance between their gaze and the target location, indicating whether they could predict where the target was going to appear. Explicit knowledge of the hidden rule was measured using post-sessions questionnaires asking about strategies used during performance.

Two types of stimulations were used. First, we applied specific spatiotemporal patterns of transcranial Direct Current Stimulation (tDCS; termed here as STAMP: SpatioTemporal Amplitude-Modulated Pattern) to tag individual memories during learning. The idea behind STAMP is to impose a unique multivariate pattern of scalp-applied currents such that the neural changes induced by this pattern get associated with concurrently encoded memories of the environment, and then reapply this pattern at a later stage to elicit or facilitate the associated memory; (see refs ^29,30^ for related prior work using oscillatory stimulation to provide a mnemonic context). In our case, some of the tagged memories were cued during the following night of sleep in parallel to up states of SW oscillation in a closed-loop manner. Other memories were only tagged during learning but not cued during sleep; and yet other memories were neither tagged during wake nor cued during sleep. Thus, a total of three tDCS stimulation conditions were compared within subjects in the same session (‘Tag & No Cue’, ‘Tag & Cue’ and ‘No Tag & No Cue’, respectively). The objective of using tDCS STAMPs with this design was to extend the TMR methodology by examining whether non-sensory information applied during wake and sleep can be used to enhance targeted memories, possibly by increasing the likelihood of their replays, similar to the enhancement achieved using auditory cues in previous studies^13,15^.

Second, we applied, only during sleep, closed-loop transcranial alternating current stimulation (tACS), coupled to SW oscillations. tACS was applied in the same group of subjects, alternating with STAMPs, through the whole night. The objective of using closed-loop tACS was to increase SW oscillations and spindle-SW coupling to improve generic consolidation of recently acquired memories (possibly by inducing a uniform increase in the quality and/or quantity of sleep replays), as recently demonstrated by us^16^ as well as others using open-loop offset oscillatory-tDCS^6,7,17^.

As control for the “Active” group of subjects (N = 12) that received both tDCS STAMP and tACS stimulations, we also ran an additional control group (N = 12) using the same protocol but administering only Sham stimulations during both wake and sleep. Thus, unlike the Active group, the “Sham” group did not have any within-subject conditions. Our hypothesis was that the application of specific tDCS STAMPs during sleep would help facilitate the individual memories they were previously associated with during wake, whereas tACS would induce a non-specific facilitation of performance for all memories, which could also lead to the gain of insight into the temporal rule. In other words, we expected ‘Tag & Cue’ memories to exhibit the biggest overnight performance improvement with contributions from both tDCS STAMPs and tACS, followed by ‘Tag & No Cue’ and ‘No Tag & No Cue’ memories with contributions only from tACS, and finally by ‘Sham’ memories with no contributions from any intervention. Moreover, in line with previous work showing memory consolidation during sleep might depend on hippocampal-to-neocortical transfer, we hypothesized that the facilitation of individual memories will be accompanied with a post-stimulation enhancement of power in the slow oscillation/delta (0.5–4 Hz) and fast spindle (12–16 Hz) bands^6^, whereas sleep-dependent insight will be associated with post-stimulation increase in the slow spindle (8–12 Hz) and beta (17–30 Hz) bands^23^.

## Results

### Explicit Detection of the Temporal Hidden Rule

There were no differences between the Active and Sham groups in any of the mood or sleep questionnaires (all *p*’s > 0.13 after correcting for multiple comparison). The post-session strategy questionnaire revealed that four of the Sham subjects and two of the Active subjects could explicitly articulate the hidden temporal rule governing the sequences already following the training sessions before sleep. Following the session after sleep, two additional Active subjects discovered the rule, but no Sham subject did (though one Sham subject articulated a wrong rule). These differences were not statistically significant (chi square test; all *p*’s > 0.6) given the limited number of subjects, but some of them were reflected in task performance for the Insight items, as shown below (see also Figs S1 and S3 in Supplementary Information for the relation between insight and task performance).

### Performance in the Task

**Figure 2:**
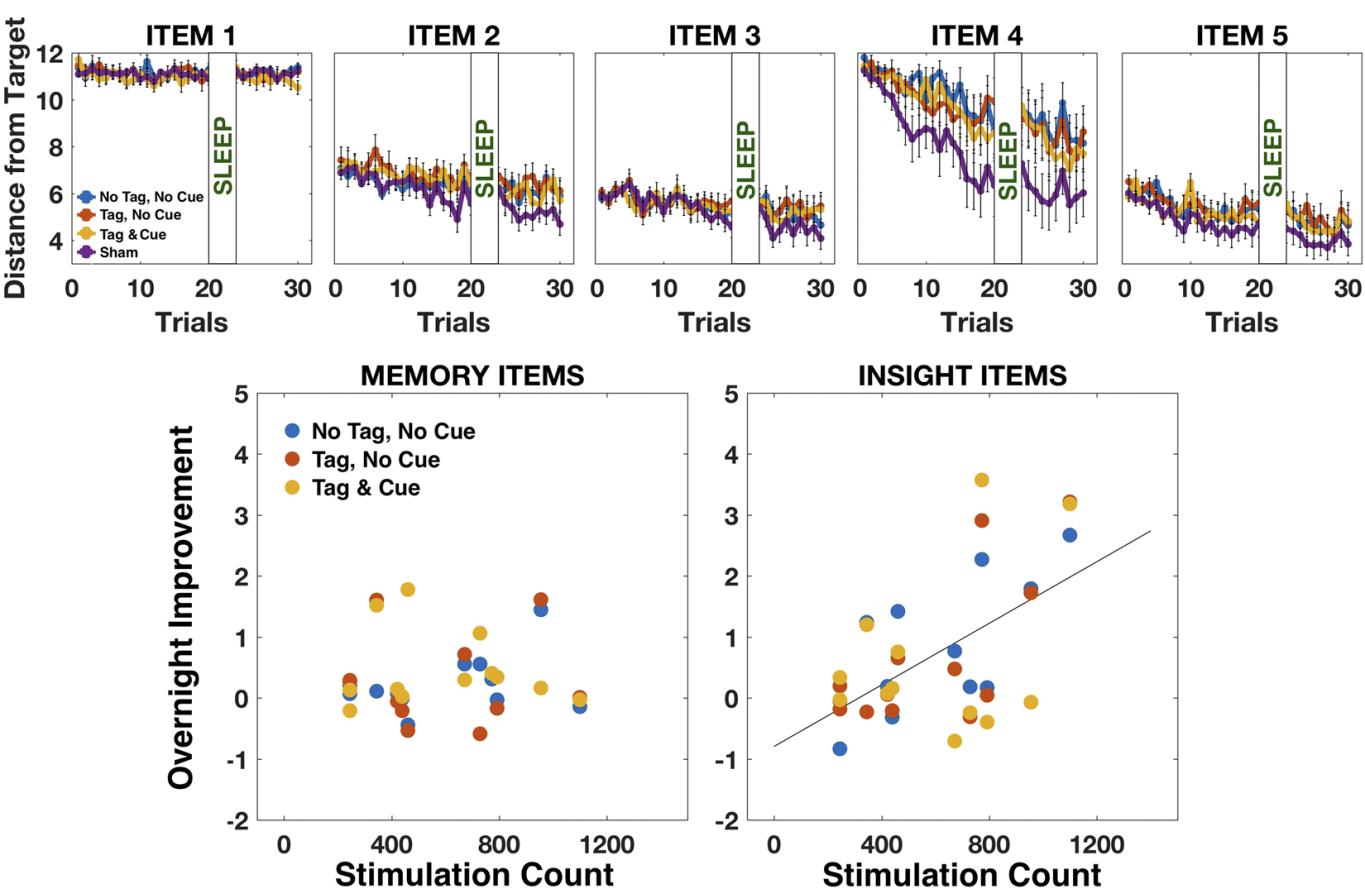
Behavioral effects in the Active and Sham groups. Upper row: Average performance for each item in a sequence for each stimulation condition within the Active group and for the Sham group. Error bars represent standard error of the mean. Lower row: Overnight performance change for the Memory and Insight items as a function of Stimulation Count. The three stimulation conditions (Tag & Cue, Tag & No Cue, No Tag & No Cue) are presented separately for comparison. Mixed-model ANOVA revealed a strong effect of Stimulation Count on overnight performance change for the Insight items (F(1,12.10) = 15.84, *p* = 0.002), but no effect on the memory items (*p* = 0.43). See text for details.

Average performance of the subjects for the Active and Sham groups for each sequence item (targets 1 to 5) is shown in Fig. 2 (upper row), with the Active group broken down by the three stimulation conditions. The only apparent difference in learning rate between the Active and Sham subjects was on item 4 before sleep, resulting from the higher number of Sham subjects having insight during training. The relatively large improvement in item 4 compared to the other items, particularly item 5, is due to its higher distance from the middle of the building; there was no difference between final pre-sleep performance on items 4 and 5 for the subjects who gained insight, with their Distance metric reaching 3.09 and 2.61, respectively, over the last 5 trials of training (*p* = 0.38, t-test). To examine the effects of stimulation during sleep on subsequent performance, we first analyzed how the Distance metric (averaged across a session for each experimental condition) was influenced by the stimulation conditions in the Active group alone. We ran a 2 × 3 × 2 mixed-model ANOVA with Item Type (‘Memory’ items 2 and 3, ‘Insight’ items 4 and 5), Condition (Tag & Cue, Tag & No Cue, No Tag & No Cue) and Time (pre-sleep final session, post-sleep session) as 3 repeated factors with a Heterogeneous Compound Symmetry covariance matrix (taken from the Materials and Methods section). To this analysis we added one covariate, the number of stimulations received during sleep – “Stimulation Count”; this number greatly varied between subjects as it depended on the detection of SW oscillations, and thus effectively determined the strength (or dose) of the experimental manipulation during sleep (since the two types of stimulations, tDCS STAMPs and tACS, were applied in an alternating manner, their number was equal within each participant. Stimulation Count was therefore computed as the total number of stimulations administered throughout the night). Results are presented in Table 1.

**Table 1:** Results of mixed-model ANOVAs in the Active group.

Effect	Num DF	Den DF	F Value	p
Type	1	11.177	7.096	**0.022**
Condition	2	88.173	1.538	0.221
Time	1	31.138	0.542	0.467
StimCount	1	10.020	0.002	0.965
Type × Condition	1	60.053	0.196	0.823
Type × Time	2	58.185	2.206	0.143
Type × StimCount	1	11.177	1.872	0.198
Condition × Time	1	71.296	0.387	0.680
Condition × StimCount	2	88.173	1.264	0.288
Time × StimCount	1	31.148	5.398	**0.027**
Type × Condition × Time	2	53.130	0.087	0.917
Type × Condition × StimCount	2	60.053	0.147	0.864
Type × Time × StimCount	1	58.185	5.014	**0.029**
Condition × Time × StimCount	2	71.296	0.378	0.687
Type × Condition × Time × StimCount	2	53.130	0.095	0.910

Note. Mixed-model ANOVA for the Active group. StimCount refers to total number of stimulations delivered during sleep. Num DF: Numerator Degrees of Freedom; Den DF: Denominator Degrees of Freedom. Significant values are highlighted in bold. See text for details.

The analysis revealed that there was no main effect of Condition on performance, nor any interactions of Condition and the other factors, indicating that the primary hypothesis stated in the Introduction section was not supported. However, there was a significant interaction between Time and Stimulation Count, indicating that the difference in performance before and after sleep was modulated by how many stimulations were delivered during sleep (F(1,31.15) = 5.40, *p* = 0.027). There was also a significant 3-way interaction of Type × Time × Stimulation Count, suggesting that the modulation of overnight performance change by the number of stimulations during sleep was different for the Insight and Memory items (F(1,58.19) = 5.01, *p* = 0.029). Finally, there was also a significant main effect of Type (F(1,11.18) = 7.10, *p* = 0.022) indicating higher values for the Insight compared to the Memory items, which is an expected effect due to the fact that memory and insight items were not balanced compared to one another in terms of the distance from the center of the building (see Materials and Methods). To follow up the 3-way interaction, we calculated the change in performance following sleep by subtracting the Distance metric after sleep from the Distance before sleep (yielding positive values for performance improvement). We then ran two separate analyses across stimulation conditions (using the same covariance matrix for repeated measures as the first analysis), one for the Insight items and one for the Memory items, with Stimulation Count as a single covariate. Results are presented in Fig. 2, lower row. The analysis revealed that for the Insight items, Stimulation Count strongly predicted the overnight performance change (F(1,12.10) = 15.84, *p* = 0.002). This correlation was driven in part by the two subjects that had insight following sleep. In contrast, for the Memory items, Stimulation Count was not correlated to the overnight change in performance (*p* = 0.43).

To compare between the Active and the Sham groups, we then ran a 2 × 2 × 2 mixed-model ANOVA with Group (Active, Sham) as between subjects factor and Item Type (Memory, Insight) and Time (pre-sleep final session, post-sleep session) as 2 repeated factors. The analysis revealed a significant main effect of Time (F(1,106.85) = 31.02, *p* < 0.001), indicating improvement in performance from before to after sleep across the two groups, and, again, a significant main effect of Type (F(1,28.99) = 12.98, *p* = 0.001). The group effect was not significant, nor any other effects.

Finally, since our experimental protocol administered stimulation every time a SW event was detected during sleep, it was possible that the Stimulation Count effect was actually resulting from the number of such SW events. To address this, we ran a control analysis on the Sham group, using the number of SW events (which were marked online by the same closed-loop algorithm used for the Active nights; see Materials and Methods) as a covariate, together with Time as a repeated factor as before. This analysis showed that there was no main effect of SW event count, nor did this factor interact with Time, indicating the number of overnight SW events did not predict overnight performance change for the Sham group (all *p*’s > 0.34).

To summarize, in contrast to our hypothesis, we found no indication of improved performance for specific sequences through targeted memory stimulation using tDCS STAMPs during sleep. However, we found that, overall, the more stimulations subjects received during sleep, the better they were able to perform the following morning on items that benefitted from the detection of a common hidden temporal rule characterizing all of the encoded sequential memories. Since subjects received stimulations that were composed of alternating tDCS STAMPs and tACS, our analysis could not differentiate which of the two was responsible for the observed effects. To address this, we next studied the neurophysiological responses to each type of stimulation during sleep in order to better identify the mechanisms that contributed to the observed behavioral findings.

### Stimulation-Induced Spectral Power Changes

**Figure 3:**
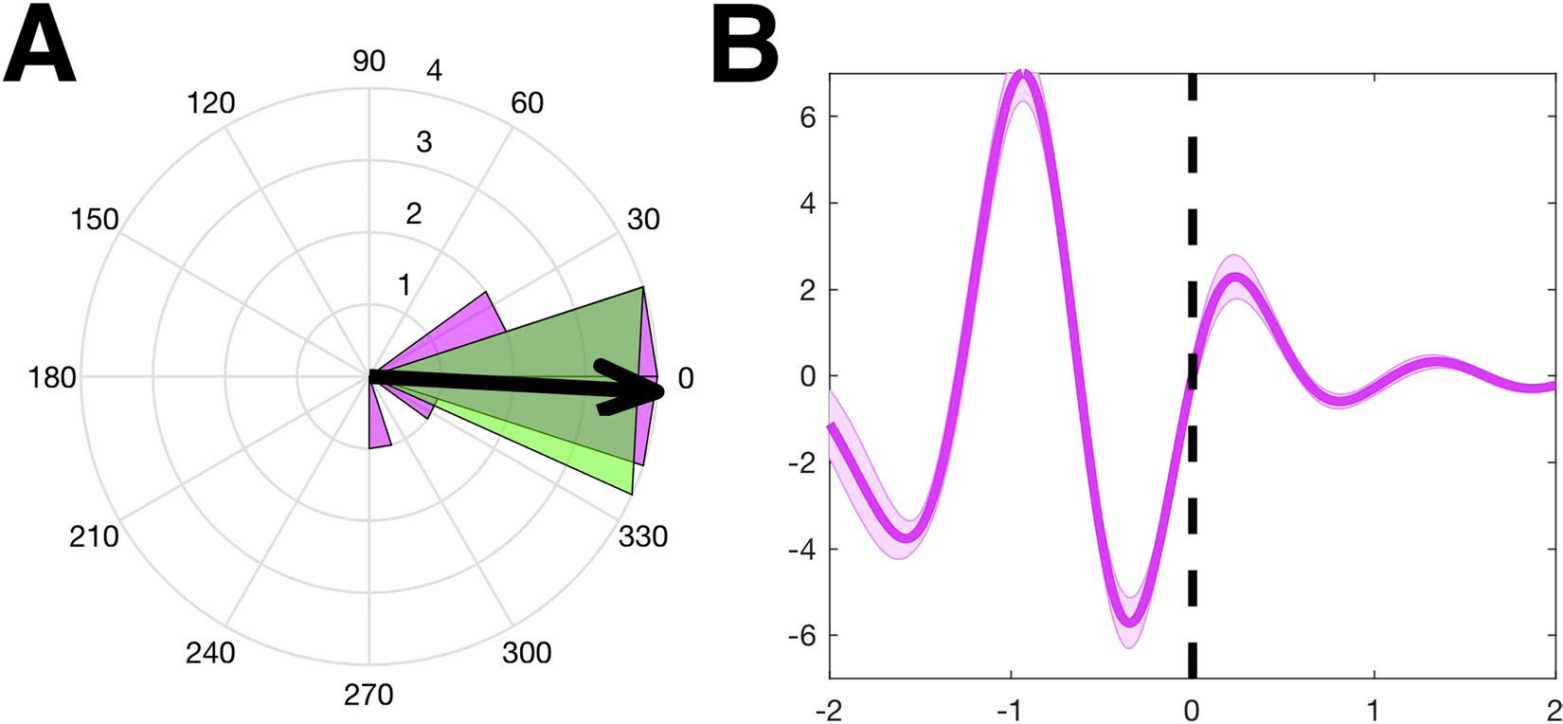
Validation of online detection of up state phases in the Sham subjects. (**A**) Polar histogram of mean phase angles across Sham subjects at the start of predicted up states. The purple histogram bars represent mean phase angles from each participant’s data, and the arrow plots the mean phase angle over these means. The dark shaded region around the arrow shows the 95% CI. (**B**) Grand mean event-related potentials (ERP) of the virtual channel centered at the start of predicted up states (time 0), averaged across Sham subjects. Data is bandpass filtered between 0.5–1.2 Hz.

To begin this analysis, we first verified the accuracy of the online SW oscillation prediction algorithm by examining the timing of the marked start of the up states. To that end, we computed the SW phase at the start of predicted up states on Sham nights (see Materials and Methods for details). Results are presented in Fig. 3. The polar histogram (Fig. 3A) shows the mean phase angles across SW events through the night for each subject (purple), as well as the mean of the mean phase angles (arrow), which is approximately pointed towards 0° (negative-to-positive zero-crossing). This was confirmed with a significant *v*-test for the mean phase angles not differing from 0° (*p* < 0.0001). Accordingly, the grand average of event-related potentials (ERP) time-locked to the start of the predicted up states across Sham subjects (Fig. 3B) reveals the presence of consistent up states indexed by the starting time-points marked online, as planned. Thus, results confirmed that stimulations in the experiment were timed using predicted and validated up states of ongoing SW oscillations. We also computed the histogram of SW events across the different sleep stages to determine in which sleep stage most of these events occurred. Results are presented in Supplementary Information, Fig. S2. In both the Active and Sham groups, the majority of stimulations (or sham stimulations) were delivered during stages N2 and N3 (SWS) compared to the other sleep stages (*p* = 0.0241 and *p* < 0.0001, for the Active and Sham groups, respectively), confirming the association between SW detection and slow oscillations.

To analyze the spectral power changes induced by sleep stimulations, post-stimulation changes in power were estimated between the Active and Sham groups using the clustering procedure described in the Materials and Methods section; in short, significant values from an independent samples comparison of relative post- stimulation power in the Active vs. Sham groups were clustered between 3 to 10 s post stimulation across all channels for five studied frequencies (SW/delta, theta, slow spindles, fast spindles, and beta; see Materials and Methods), separately for tACS and STAMP stimulations. The significant spatiotemporal clusters of these *t*-values, as determined by permutation test, were defined as the “contrast clusters”. A similar cluster analysis was then carried out when correlating overnight changes in performance with the spectral power changes following stimulation, separately for the Active and Sham groups. This analysis was restricted by using the significant contrast clusters as masks to sub-select the channel × time bins that showed a significant change in spectral power. This way we were able to associate overnight performance changes with specific spectral power modulations induced by either simulation.

**Figure 4:**
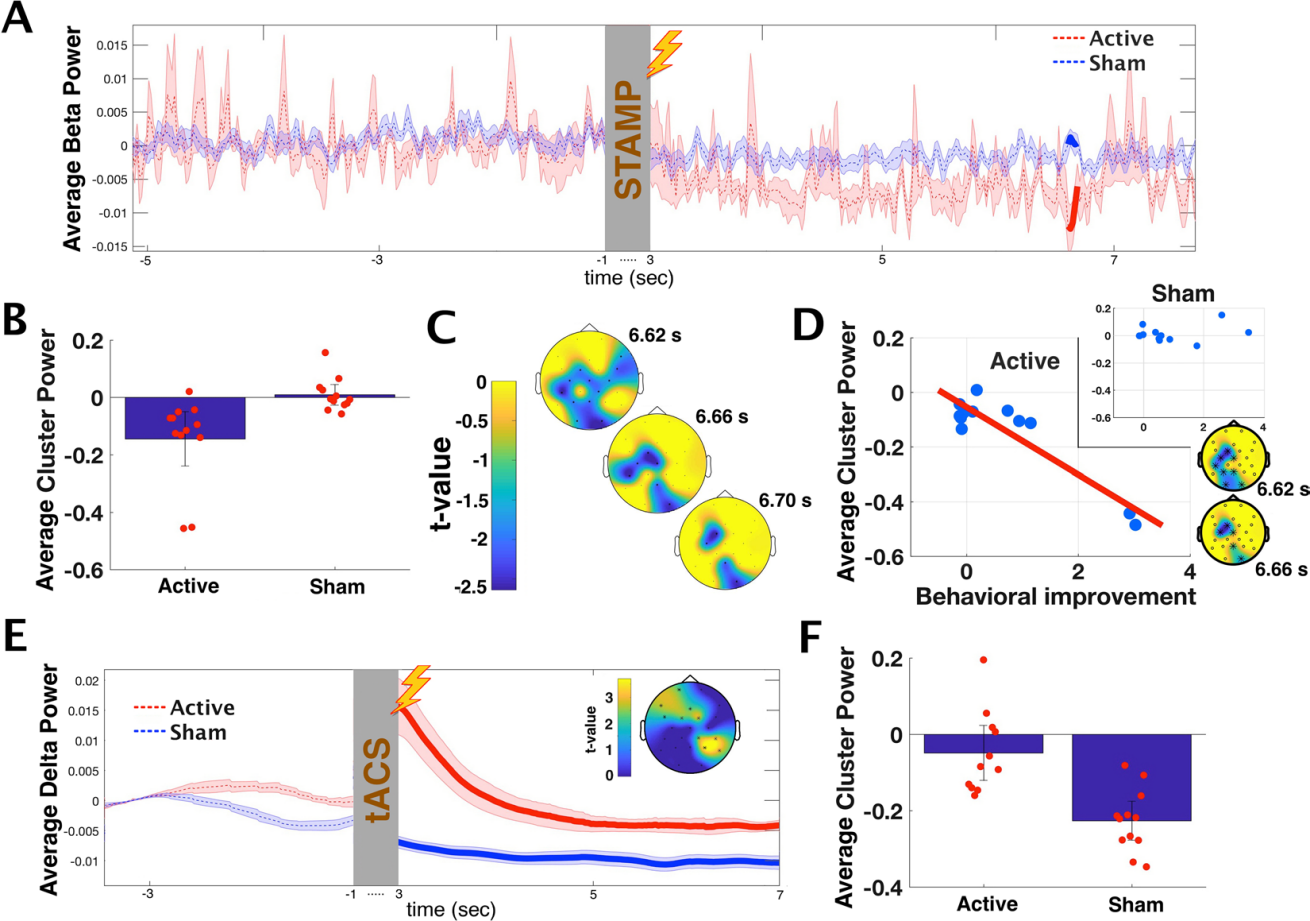
Effects of Sleep Stimulations. (**A**) Average beta power (17–30 Hz) as a function of time before and after STAMP stimulation for the Active and Sham groups. The time period from 1 second prior to stimulation onset to 3 seconds following stimulation offset, indicated by the gray bar, is trimmed from the display to avoid showing stimulation-related voltage artifacts. Values are centered around the pre-stimulation means. Time bins where the difference between the groups reached statistical significance are bolded. (**B**) Average beta power of the contrast cluster with the significantly different time bins for the Active and Sham groups. Bonferroni-corrected clusterwise *p* = 0.035. Error bars are standard error of the mean. Red dots represent individual subjects. (**C**) Scalp topography of the t-values for the relative power changes in the significant contrast cluster, at three times points within the significant window. Four channels participated in the cluster throughout, and twelve in total, marked by asterisks. (**D**) Correlation of relative power change and overnight performance change in the Distance metric for the insight items, across Active subjects (primary; Clusterwise *p* = 0.008) and Sham subjects (inset; n.s). Also displayed is the scalp topography of the correlation cluster at two time points. (**E**) Average delta power (0.5–4 Hz) as a function of time before and after tACS for the Active and Sham group, similar to (**A**). All time bins from 3 to 10 s following stimulation were significantly different (bolded; the full extent from 3 to 10 s is not shown to save space). Inset shows representative scalp topography of the t-values of the relative power changes in the cluster (5 s after stimulation offset, when the curves became stable). (**F**) Average delta power of the cluster with the significantly different time bins for the Active and Sham groups. Bonferroni-corrected clusterwise *p* = 0.008. Error bars are standard error of the mean. Red dots represent individual subjects.

The analysis revealed that for STAMP stimulation, one cluster in the beta power band (17–30 Hz) differentiated between the two subject groups, such that the power in this cluster decreased following the stimulation in the Active group more than in the Sham group (Fig. 4A,B). This cluster temporally extended from 6.62 to 6.72 s relative to stimulation offset, and had a Bonferroni-corrected clusterwise *p* value of 0.035 (*p *= 0.007 uncorrected). Scalp topography of *t*-values for the relative power changes in the cluster indicated an early distribution over wide areas, which then narrowed to four channels over occipital, left parietal, and left frontal regions (channels FC1, C3, O2 and Pz in the 10/10 system; see Fig. 4C). This topographical pattern was unrelated to the topography of the four STAMPs that were used (see Fig. S4 for details), indicating it was not a simple function of the stimulation. As can be seen in Fig. 4A, the reduction in beta power for the Active group, albeit not reaching statistical significance for the entire time window using the permutation-based statistical test, was numerically evident throughout. To confirm this, we carried out a follow-up test by extracting the average beta power in the four channels that were persistent throughout the 100 ms duration of the significant contrast cluster (namely, FC1, C3, O2 and Pz), and then compared Active and Sham power over those channels for the full 3 to 10 s duration post stimulation. We found that the Active subjects showed significantly less beta power as compared to the Sham subjects (t(22) = −3.48, *p* = 0.0021), indicating beta power differentiated between the groups over these channels for the entire time window following stimulation.

Next, we correlated the overall overnight changes in the Distance metric with changes in spectral power within this contrast cluster across the Active subjects. We found a significant sub-cluster between 6.62 and 6.68 s, with a clusterwise *p* value of 0.009, indicating that the stronger the stimulation-induced decrease in beta power had been, the higher was the behavioral improvement. In contrast, no significant correlation clusters were found for the Sham subjects. We then repeated the analysis for the Active group separately for the improvement in Insight and in Memory items. For the Memory items, no significant clusters were found. Conversely for the Insight items, we again found a significant sub-cluster between 6.62 and 6.72 s, with a clusterwise *p* value of 0.008 (Fig. 4D). As can be seen in the figure, this effect seems mostly driven by the two subjects that had insight (the two lower-right data points). To examine whether the effect survives when the extreme values of these two insight subjects are diminished, we ran a post-hoc test computing the Spearman’s Rank correlation between the individual cluster strength and the improvement in Insight items. We found that the correlation remained significant at a trend level (r = −0.5245, *p* = 0.083), indicating that it did not entirely rely on the seemingly outlier values of the two insight subjects.

Repeating the analysis for tACS, we found a cluster in the delta power band (0.5–4 Hz) that differentiated between the Active and the Sham groups, such that the power in this cluster decreased less following the stimulation in the Active group compared to the Sham group (Fig. 4E,F). This cluster temporally extended from 3 to 10 s relative to stimulation offset (i.e., the full time window we examined post stimulation), and had a Bonferroni-corrected clusterwise *p* value of 0.008. This result confirmed that our application of tACS at SW frequency had the expected effect of enhancing endogenous slow waves and delta power (see Materials and Methods), replicating previous results with tACS^16^ and offset oscillatory-tDCS^6^. However, correlational analysis revealed no sub-clusters that were associated with any overnight performance changes in either the Active or the Sham group.

To summarize, the clustering analysis suggested that STAMP stimulations during sleep were more likely responsible for the overnight facilitation in performance for the Insight Items in the Active group subjects, and did so by reducing beta power activity. While tACS also had an effect – namely, preventing power reduction in the delta band activity – this did not directly affect behavior in the task.

## Discussion

Our main findings in the current study are threefold. First, we showed that transcranial current stimulations during SW oscillations are capable of influencing the discovery of hidden rules governing encoded memories. Specifically, we showed that after first being exposed to repeating sequential events in the environment while receiving STAMP stimulations, subjects became more sensitive to the repeating temporal pattern in those sequences at a second session following sleep as the number of reapplications of the same STAMP stimulations, together with additional non-specific tACS, increased throughout the sleep period. Further, this relation was shown to correlate with STAMP-dependent modulation of beta power. This result confirmed our hypothesis that transcranial stimulation during sleep could facilitate high-level cognitive functions beyond simple, veridical memory consolidation. Second, unlike our hypotheses, we found no evidence for targeted memory consolidation in which only memories that were both tagged and cued by STAMP stimulations show improved performance overnight. Finally, we found no significant behavioral difference between Active and Sham groups in terms of average task performance, though the stimulations did lead to differences between the groups in the power of specific frequency bands; indeed, the association we found between beta power and performance was only significant for the Active group.

The results we obtained raise several questions. First, considering the Active versus Sham groups, it is unclear why no differences in performance were obtained following sleep. In comparison, closed-loop auditory TMR during naps was recently shown to enhance overall navigation performance in virtual environments compared to controls, with concomitant changes in fast spindle power following TMR cueing (though neither dose effects nor specificity of the TMR effect to individual locations on the map was demonstrated; 18). One possible reason for this discrepancy is that transcranial current stimulation to the brain during sleep might be ineffective in manipulating memory consolidation. Indeed, since the original demonstration of declarative memory enhancement using offset oscillatory-tDCS during sleep^6^, several attempts have failed to replicate or extend those findings^31,32^, with some recent studies suggesting that transcranial current stimulation at the typical safe levels (<2.5 mA) may be too weak to have significant effects on brain oscillations and neuronal spiking in humans, due to shunting and attenuation of the scalp-applied currents by skin and skull^33,34^. However, those previous studies^6,31,32^ applied open-loop stimulation. In contrast, using a closed-loop tACS protocol during sleep, we recently demonstrated significant modulation of SW spectral power and SW-spindle coupling, resulting in a significant behavioral effect related to memory generalization^16^. Moreover, cathodal oscillatory-tDCS applied at specific frequencies during sleep was recently shown to modulate declarative memory for neutral and emotional words in humans^35^ and, in a non-human primate study, we showed that 2 mA tDCS targeting the prefrontal cortex could lead to improved learning performance resulting from changes to inter-areal coherence in gamma frequencies^36^. Therefore, it is unlikely that our null results indicate an inherent inability of transcranial current stimulation to modulate memory consolidation. A more likely explanation, especially given our findings of a dose effect of stimulation on insight learning within the Active group alone, is a lack of experimental power. When comparing the subjects within the Active group, the number of stimulations received by each subject could be used as a factor that reflects the strength of the manipulation, thus allowing to account for small resulting differences in overnight performance changes. In contrast, when comparing the performance between the Active and Sham groups, the variable being tested is the average value across the subjects; and since the manipulation led to only two Active subjects (out of N = 12) gaining clear insight into the hidden rule following sleep, their influence on the average effect is likely not strong enough to pass a significance test compared to the Sham group. Note, however, that given the larger improvement in performance on item 4 and the higher number of subjects gaining insight in the Sham compared to the Active group during training, it is possible that tDCS STAMPs during training could, by themselves, hamper insight learning prior to sleep (Fig. 2, item 4; Fig. S1). Though not the focus of the current study, this unexpected effect should be further investigated in future work.

A second question raised by our results considers the Active group alone. It is not clear why only the Insight items were modulated by stimulation and why there were no differences among the three stimulation conditions (namely, Tag & Cue, Tag & No Cue, No Tag & No Cue). Below we consider three possible hypotheses that could potentially account for this pattern of findings.

First, it is possible that tDCS STAMPs during the night did preferentially benefit the specific sequences belonging to the Tag & Cue condition as we hypothesized, but the effect was, on the one hand, too weak to be evident for the Memory items, and, on the other hand, blurred for the Insight items because of their shared hidden rule such that the three stimulation conditions looked similar. The effect could have been too weak for the Memory items because those were composed of the 2^nd^ and 3^rd^ targets in each sequence; therefore, when trying to memorize a specific sequence, the 2^nd^ item could only be strengthened by a previous activation of the 1^st^ item, while the 3^rd^ could only be strengthened by the 1^st^ and 2^nd^ items. Consequently, consolidation of these items through Memory replay was constrained to the strengthening of fewer associative links to begin with. In contrast, the Insight items, even when disregarding the hidden rule, were always composed of the 4^th^ and 5^th^ targets in each sequence and could therefore be strengthened by all of the items preceding them in that particular sequence. Consequently, they had more potential associative links that could be strengthened through replay compared to the Memory items. This difference alone could have led to the observed facilitatory effect for the Insight items while yielding a null result for the Memory items. In addition, due to the common hidden rule governing the Insight items across the stimulation conditions, learning the predictive structure for these items in one sequence could have quickly led to the realization of the hidden rule and thus for this knowledge to propagate to the other sequences. Indeed, individuals that gained insight did show a sudden improvement for the Insight items of all sequences within a few trials, and the correlation in performance across the three stimulation conditions was highly and significantly positive for the Insight items (but less so for the Memory items; see Supplementary Information, Fig. S3). Such propagation of knowledge could therefore blur the distinction among the stimulation conditions following sleep, and appear as if STAMPs helped all conditions rather than just the Tag & Cue condition.

Nevertheless, this account may be unlikely. First, if indeed the Tag & Cue sequences were preferentially strengthened during sleep, leading subjects to notice the hidden structure and propagate that knowledge to the other sequences (thus blurring the Condition effect when looking across the full post-sleep session) we would still expect the Insight items in the sequences belonging to the Tag & Cue condition to improve a little earlier than those in the sequences of the other conditions when inspected on a trial-by-trial basis. However, as can be seen in Fig. 2 (upper row), such a clean trend does not seem to exist. Second, the effect of STAMPs on Insight items was mediated by the modulation of beta oscillations, previously shown to relate to insight learning (27; see discussion below), and not through slow and spindle oscillations as expected from previous studies of targeted memory consolidation^13–15^. As such, there is no conclusive evidence for the targeted activation of sequences by tDCS STAMPs in our experiment.

A second hypothesis accounting for our results assumes that STAMP stimulations during wake did not induce any tagging effects, but STAMP stimulations during sleep created some kind of a non-specific facilitation in memory consolidation, possibly through a general increase in replay, as originally assumed for tACS. While we cannot refute this possibility, it does not seem likely either. If no tagging occurred, both tACS and STAMPs during sleep could have potentially contributed to such non-specific consolidation of memories. Previous findings in the literature have already linked non-specific tACS during sleep to memory consolidation^16,37^. In contrast, in our results, tACS did not elicit any spectral power changes within its post-stimulation period that were correlated with behavior, whereas STAMPs did. Therefore, it stands to reason that the one unique feature of STAMPs compared to tACS in our experimental protocol – their application in the previous waking period – had something to do with their facilitatory effect.

A third, perhaps more likely hypothesis, is based on the possibility that the STAMP stimulations, instead of tagging only the specific sequences during which they were applied, could have actually tagged the entire learning session. In other words, applying STAMPs during learning could have associated the stimulation patterns with the learning context, rather than with any specific stimuli. Then, at sleep, reapplication of some of those STAMP patterns would have biased sleep-related memory consolidation towards task-related memories as a whole by virtue of reactivating the learning context (cf., 14). Consequently, all sequences would have been reactivated in equal probabilities rather than just the ones belonging to the Tag & Cue condition, hence not giving any particular condition an advantage over the others. Further, Insight items are likely to be the chief beneficiaries of such a process since the hidden rule could be extracted from any of the sequences whereas each memory item could only be facilitated by reactivation of the specific sequence it belonged to – which, under this hypothesis, would comprise of only one-sixth of the potential task-related reactivations. Consequently, STAMP stimulations may very well have benefitted Insight items of all sequences, but none of the Memory items in any sequence.

Assuming all sequences were reactivated following STAMP stimulations, how could that facilitate insight into the hidden rule? As discussed in ref.^28^, past findings concerning sleep-induced insight are often characterized by a hidden rule having a temporal structure: event X at time *t* predicts event Y at some time-point in the future, typically a few seconds later. This type of hidden regularities are not easily identified in real time; however, during SWS, endogenous reactivations of these experiences, occurring in a time-compressed manner^1,38^, essentially bring event X and Y closer together in time and could thus allow Hebbian mechanisms – or other activity-dependent local plasticity processes in the hippocampus – to more readily associate them directly^39,40^. These associations can potentially form the basis of conscious identification of the hidden rule by the prefrontal cortex when performing the task again the following morning. Therefore, assuming STAMP stimulations during the night bias replay towards task-relevant sequences rather than the many other events subjects could have experienced on or just prior to the experimental day, more opportunities for forming the critical associations (in our case, the connection from the first item to the fourth and from the third to the fifth due to the A-B-C-A-C structure) would arise as a result.

Our findings suggest that the effect of STAMPs on insight was mediated by a decrease in beta power following stimulation. Both human and rat studies indicate that beta activity during SWS is coupled to slow oscillations such that beta power increases during up states and decreases during down states, similar to spindles^41,42^. It is therefore likely that beta oscillations are part of the hippocampal-cortical dialogue during SWS supporting memory consolidation. Nevertheless, the distinguishable role that beta oscillations play during sleep compared to other frequency bands remains unclear. Reduction in beta power during wake has been linked to active engagement of cortical modules during retrieval of episodic memories^43^. If the same mechanism also operates during sleep, our findings could indicate that STAMPs contributed to memory recall. Importantly, beta oscillations during SWS have been previously identified as markers of insight into temporal patterns^27^. Specifically, Yordanova and colleagues have shown, using the Number Reduction Task (with no stimulation), that subjects who gained insight following sleep had a general increase in the beta band power during SWS compared to subjects who did not gain insight. In our study, insightful subjects had a decrease in beta power rather than an increase. While the reason for this discrepancy remains a matter of speculation, one possibility is that the beta power reduction in our results actually indicates a refractory period following a stimulation-induced increase. Indeed, using auditory stimulations administered at the peaks of endogenous SW oscillations during SWS, Ngo and colleagues^44^ reported stimulations caused an initial increase in SW and spindle power when compared with sham, followed by a decrease that was likely due to refractory mechanisms. Additional results from our group using closed-loop slow-wave tACS during sleep corroborate these findings, with SW power first increasing and then decreasing in response to stimulation (16; see also Fig. 4E for a similar trend). Since a reliable EEG signal was not available for the first 3 s after stimulation offset in the current study, we cannot determine with certainty what the effect on beta within this time window has been – especially given that changes in beta power within those first 3 s would have left fewer residuals than changes in SW oscillations, even if occurring at the same time, simply due to the differences in temporal uncertainty in estimating the two bands based on their frequency ranges. Nevertheless, a close inspection of Fig. 4A does hint that the decrease in beta power develops rather than already exists once the EEG signal becomes readable after the stimulation offset. Therefore, it seems plausible that the observed beta power reduction in our results actually represents a refractory response following an initial STAMP-induced increase, consistent with the previous results linking insight learning and beta power increase during SWS^27^.

The reduction in beta power was initially widespread over the scalp, but then narrowed down to be more pronounced on the left hemisphere (Fig. 4C). This resonates with previous investigations of neural correlates of insight during sleep, indicating the involvement of a wide network of regions, from occipital to frontal, that is largely lateralized^45^. However, the typical finding is that of increased beta power on the right hemisphere rather than decreased activation on the left. This apparent contradiction might be resolved if we consider that unlike other studies, we administered a stimulation protocol during sleep. Indeed, additional non-sleep studies show that anodal tDCS to the left prefrontal cortex facilitates insight learning, possibly due to the modulation of executive control^46,47^. It therefore seems that there might be two routes to insight: Naturally-occurring, indexed by increased right hemispheric activity; and stimulation-induced, indexed by modulation of activity in the left hemisphere.

It is important to reiterate that the relation between beta modulation and behavioral facilitation was mostly driven by the two subjects that had insight following sleep (Fig. 4D). While the effect remained significant at a trend level even when diminishing the extreme values of these insight subjects using Spearman’s Rank correlation, it remains possible that rather than reflecting modulation across all participants, the beta power change mostly differentiates between insight and non-insight subjects. In that case, small beta modulations, as the ones seen in the 10 subjects who did not have insight, are not predictive of behavioral changes. Since we only had two subjects gaining insight following sleep, the current dataset is too small to distinguish between the two interpretations.

Finally, there remains the question of why, despite successfully replicating previous findings showing stimulation-induced increase in SW oscillation power during sleep with open-loop offset oscillatory-tDCS^6,7,17^ and closed-loop tACS^16^, we failed to replicate the facilitatory effects of this increase on behavioral performance? One possible answer is that previous studies employed relatively simpler tests of declarative memory, such as recall of paired associates^6,7,17^ and memorization of target locations^16^. It is possible that consolidation of different memory types rely on somewhat different oscillatory behavior. For example, Lustenberger and colleagues^37^ found that tACS at spindle frequencies affect procedural memories but not declarative ones. Since our task was the first to use sequential memories with both declarative and procedural elements in combination with transcranial stimulation during sleep, it is possible that SW enhancement did not tap into the optimal frequency bands required for the associated performance facilitation. A second possibility is that our alternating usage of tDCS STAMPs and tACS interfered with each other, thus abolishing the beneficial effects of SW enhancement.

To conclude, the current study is the first to demonstrate that transcranial stimulation during sleep can facilitate a high-level cognitive process such as insight learning. However, questions remain regarding the minimal requirements for the stimulation that drives the improvement in insight-related performance and the memory consolidation mechanisms that are consequently modulated. Future studies will need to address these questions with higher number of participants, focusing on a single type of stimulation. For example, such future studies could use only tDCS STAMPs, with or without earlier tagging during wake, to examine their effect on learning in isolation; or, alternatively, administer only tACS in the beta band to directly examine its effect on insight learning.

## Materials and Methods

### Participants

A total of 24 healthy participants (14 females; mean age 21.25 ± 4.67) were recruited using flyers placed around campus of the University of New Mexico and surrounding community and completed the experiment for monetary compensation. Inclusion criteria included the use of English as a first language, completion of high school, and no history of head injury with loss of consciousness for longer than five min, neurological or psychiatric disorder, or of alcohol or drug abuse. In addition, participants were all right-handed according to the Edinburgh Handedness Inventory^48^, were non-smoking, had no excessive alcohol (<10 standard drinks/week) or caffeine (<24 oz/day) consumption, were not currently taking any medication significantly affecting the central nervous system, had no implanted metal, had no sensitivity or allergy to latex, had good or corrected hearing and vision, and reported no sleep disturbances. Women who were pregnant, or thought they may be, were excluded. All participants provided signed informed consent to participate in the study, which was approved by the Chesapeake Institutional Review Board. All research was performed in accordance with the relevant guidelines and regulations.

### Behavioral Methods

#### Behavioral task and stimuli

The behavioral task used in this study measured learning of items in various temporal patterns, differentiating between those that can benefit from insight into a hidden temporal rule and those that don’t. Going beyond common approaches in the field, which often use simplistic stimuli delivered discontinuously on a trial by trial basis, we aimed to integrate the behavioral measurements into naturalistic settings, both to add ecological validity to our findings as well as to create an engaging experience that could potentially increase the likelihood of encoded memories being consolidated during the ensuing sleep period. To that end, we developed a novel realistic task, administered in a 3D environment using virtual reality goggles (HTC Vive, HTC Inc.) that simulated a “surveillance” operation in a Middle-Eastern-looking city. Within this virtual environment, subjects were situated on a porch, facing a building with 20 windows where characters were passing by, one at a time (Fig. 1; see also Movie S1 in Supplementary Information for a video demonstration). Their objective was to “take a picture” of each and every character (hereafter, “target”) by orienting a crosshair in their head-mounted virtual reality display towards the target – as though they were pointing a camera – and pressing a button on a hand-held controller.

Targets were presented in set sequences, with each sequence including 5 targets appearing one after the other in distinct windows. Each target was visible for 1 s randomly placed within a 2-s time window, followed by the next target in the sequence, making the length of a sequence vary between 8 to 10 s (see Fig. 1). There was a variable Inter-Stimulus Interval (ISI) of 6–8 s in between sequences. In addition to the targets, the VR environment also included distractors occurring randomly throughout the task such as background noise, cars and helicopters passing by, as well as lights, fans and blinds turned on and off in windows when they were not used as part of a sequence. Subjects’ experience was thus continuous, with no indication of a “trial” structure other than the temporal interval between sequences.

Six distinct sequences were included in the task. Each sequence was comprised of its own set of windows where targets could appear, but sequences were balanced in terms of the distance of the windows from the center of the building, in the following way: The six sequences were divided into three groups of 2 sequences each, such that for each of the positions in the sequence (1 to 5), the distance from the target’s window to the center of the building, averaged over the two sequences of one group, was approximately equal to that of the other groups (the balancing was performed with respect to the center of the building because subjects, at the early stages of learning in this task, always tend to return their head position to the center after responding to a target – an optimal strategy given the symmetrical arrangement of the windows around the center, as indicated in Fig. 1. Note, however, that the distance of each position in a sequence was not balanced with the other positions across sequences, due to the limited number of possible sequence arrangements without repetition). Crucially, all sequences also adhered to a fixed, hidden temporal regularity in the locations of the last two targets (see Fig. 1): While the first three targets of each sequence appeared in three different windows (A, B, C), they were followed by two targets appearing again in windows A, C. Thus, each sequence was always comprised of windows in the order A, B, C, A, C. Each experimental session included a total of 60 sequences, with each of the six distinct sequences appearing 10 times in a mixed, random order. Before each session, subjects were instructed to take a picture of each target as quickly and as accurately as possible, but they were not informed of the common regularity underlying all sequences. There were two training sessions before sleep and one test session after sleep. During performance on a training session, feedback was given as follows: first, the crosshair changed color to red if the button was pressed too far from the target; to yellow if the target was captured successfully but later than 600 ms after onset; or to green when captured successfully within less than 600 ms. Second, a grunting sound was heard if a target was completely missed. The test session was identical to training, but no feedback was given.

#### Design

Two independent groups of N = 12 subjects were run, one with active stimulation during wake and sleep and one with sham stimulation. Sham subjects were treated the same as Active subjects with the exception that no stimulation currents were applied either during waking or sleep. We employed a within-subjects design for the Active group to compare, within a single night, three stimulation conditions (namely, ‘Tag & Cue’, ‘Tag & No Cue’, and ‘No Tag & No Cue’), to which the six sequences were assigned in subsets of two each. For each Active subject, two sequences were assigned to each condition, preserving the paired groupings that balanced the distance of targets from the center of the building. Across subjects, the possible assignments of the sequence pairs to stimulation conditions were counterbalanced. During training, the presentation of the two sequences belonging to the ‘Tag & Cue’ condition was accompanied with distinct concurrent tDCS montages (i.e., STAMPs; see below for details), aimed to create an association between each of those two sequences and their corresponding STAMPs (thus ‘tagging’ them); then, during sleep, the same STAMPs were applied, attempting to ‘cue’ the learned associations and thus facilitate the consolidation of those sequences. The sequences belonging to the ‘Tag & No Cue’ condition also received specific tDCS STAMPs during learning, but without additional stimulation of those STAMPs during sleep. Thus, this condition served to differentiate between the effects of stimulation during wake compared to the combined effects of stimulation during both wake and sleep. Finally, the sequences belonging to the ‘No Tag & No Cue’ condition never received any STAMP stimulation. The assignment of the 4 STAMPs to the sequences in ‘Tag & Cue’ and ‘Tag & No Cue’ conditions was randomized for each subject, but counterbalanced such that all combinations appear in an equal amount across subjects. No STAMPs were applied for the Active group in the test session. Finally, in addition to the tDCS STAMPs, subjects in the Active group also received closed-loop tACS during sleep (described in detail below), which was not associated to any individual encoded memory. The ‘Tag & No Cue’ and ‘No Tag & No Cue’ conditions served to differentiate the effects of tACS from those of combining tACS and STAMPs.

#### Experimental procedure

The experimental procedure, conducted over the course of three days, included two nights spent in the sleep laboratory: an adaptation night, followed by an experimental night. Figure 1 depicts the protocol schedule used for each subject. Participants were randomly assigned to one of the two groups (Active, Sham).

During the adaptation night, participants arrived at the sleep laboratory and were first exposed to the virtual reality (VR) environment and task procedures via a short practice session, where they viewed a short habituation sequence designed to familiarize them with the VR environment and picture taking procedures. They were then prepped for polysomnographic (PSG) recordings during sleep. Lights out occurred between 22:00–23:00, and participants were allowed to sleep for up to 8 uninterrupted hours before being awoken. Upon waking, participants filled out the Karolinska Sleep Diary (KSD; ref.^49^) to assess subjective sleep quality.

Subjects then returned to the laboratory at approximately 19:00, and were set up with the apparatus that collected EEG data and delivered stimulation (see below). They first completed a brief baseline mood questionnaire to assess potential effects of STAMP stimulation on subjective mood. The mood questionnaire was derived from several items in the Positive and Negative Affect Schedule (PANAS^50^) and consisted of nine questions on a 0–5 Likert scale. Items included feelings of nervousness or excitement, tiredness, confusion, sadness, degree of frustration, dizziness, nausea, degree of physical pain or discomfort, and ability to pay attention. Next, we administered the physical sensation questionnaire, which assessed subjective sensations due to STAMP stimulation. Following the questionnaires, subjects sat in front of the computer, put on the HTC Vive^®^ headset, and heard the task instructions. Two training sessions were administered, each lasting approximately 20 min with a 15 min break in between. The task was administered while subjects were connected to the EEG/tCS device, and STAMP stimulation (or sham) was applied during training as detailed later. After each training session, participants rated three types of sensations (itching, heat/burning, and tingling) on a 0–10 Likert scale, where 0 indicated no sensation at all and 10 indicated the most intense possible sensation. Any report of a seven or above resulted in immediate cessation of stimulation and termination of the experiment, without penalty to the participant. No subjects were lost due to high sensation ratings or being uncomfortable. Finally, participants completed an exit mood questionnaire, identical to the entrance questionnaire, as well as a questionnaire assessing their strategy for executing the task (to detect whether they explicitly report noticing any patterns in the target appearances).

Participants were then prepped for PSG recordings and went to sleep between 22:00–23:00. Rise time, accordingly, was between 06:00–07:00. During sleep, a trained research assistant monitored streaming EEG data and started the tACS/STAMP intervention at 4 min of continuous visible N2/N3 sleep and allowed the automated closed-loop algorithm, described in details below, to run through the remainder of the night. Active stimulations were 3.0 mA tACS (1.5 mA/hemisphere) and 2.5 mA tDCS STAMPs. The closed-loop intervention system was paused if the participant showed signs of waking and resumed after the participant was again in N2/N3 sleep. Upon waking, participants filled out the Karolinska Sleep Diary (KSD) to assess subjective sleep quality. Finally, they completed a test session of the task and again filled out the strategy questionnaire, after which they were disconnected from the EEG/tCS hardware, and released.

#### Analysis

To measure subjects’ knowledge of the sequences we computed the *Distance from target* as the main performance metric. Specifically, upon each target’s onset, we recorded the distance (in arbitrary units relative to the display size) between the location of the crosshair, indicative of where the subject’s head was pointing at, and the target’s location. This metric returns low values (potentially even 0) when subjects know exactly where the next target would appear, and higher values when subjects have little or no knowledge of the location of the upcoming target. To differentiate among targets that could be predicted following insight into the hidden temporal structure and those that can only be learned by memorizing the individual sequence they belonged to, we computed the Distance metric separately for the average over targets 4,5 (‘Insight’ items) and for the average over targets 2,3 (‘Memory’ items). Target 1, the first in each sequence, could never be predicted (due to the random ordering of the sequences) and was therefore not considered. Two behavioral analyses were performed. First, within the Active group, a 2*3*2 mixed-model Analysis of Variance (ANOVA) was performed, with Item Type (Memory, Insight), Condition (Tag & Cue, Tag & No Cue, No Tag & No Cue) and Time (pre-sleep, post-sleep; pre-sleep refers to the last training session before sleep) as 3 repeated factors with a Heterogeneous Compound Symmetry covariance matrix (chosen for having the lowest AIC score compared to other possible covariance matrices, though results did not depend on this choice), and number of stimulations received during sleep as a covariate. Second, across groups (with values for the Active group averaged across conditions), a 2*2*2 mixed-model ANOVA was performed with Group (Active, Sham) as a between-subject factor and Item Type and Time as 2 repeated factors,. Additional control ANOVAs were performed as well, as described in the Results section. Statistical analyses were carried out in SPSS 22 using the linear mixed-model procedure.

### Electrophysiological Methods

#### Apparatus

32-channel physiological data collection and simultaneous 32-channel stimulation were conducted using the StarStim64 device (Neuroelectrics, Inc.). The 64 electrodes were held in place using a neoprene head cap, according to the international 10/10 system (recording: *P7*, T7, *CP5*, *FC5*, *F7*, F3, *C3*, *P3*, *FC1*, *CP1*, *Pz*, PO4, *O2*, Oz, *O1*, PO3, *CP2*, *Cz*, *FC2*, *Fz*, AF3, *Fp1*, *Fp2*, AF4, *P8*, T8, *CP*6, *FC6*, *F*8, F4, *C4*, *P4;* stimulation: O10, TP8, P6, PO8, FT8, F6, C6, FC4, CP4, C2, P2, AF8, F2, Fpz, FCz, AFz, F1, AF7, Iz, POz, P1, CPz, C1, CP3, FC3, C5, F5, FT7, PO7, P5, TP7, O9). Solidgeltrodes (NE028, Neuroelectrics, Inc.) and pistim electrodes (NE024, Neuroelectrics, Inc.) were used for physiological data collection and stimulation, respectively. EEG data was collected from 23 of these 32 sites (marked in italics above). The remaining electrodes (P03, P04, Oz, AF3, AF4, F3, F4, T7, T8) were used to record EOG, EMG, and ECG to allow for the detection of artifacts and sleep stages, or were discarded during post-hoc analysis due to proximity to tACS stimulating electrodes. The physiological data was sampled at 500 Hz. Common Mode Signal (CMS) and Driving Right Leg (DRL) reference electrodes (stricktrodes: NE025, Neuroelectrics, Inc.) were placed on the mastoids. No online hardware filtering, except line noise (60 Hz) filtering, was applied during collection.

#### Waking transcranial direct current stimulation (tDCS) STAMPS

**Figure 5:**
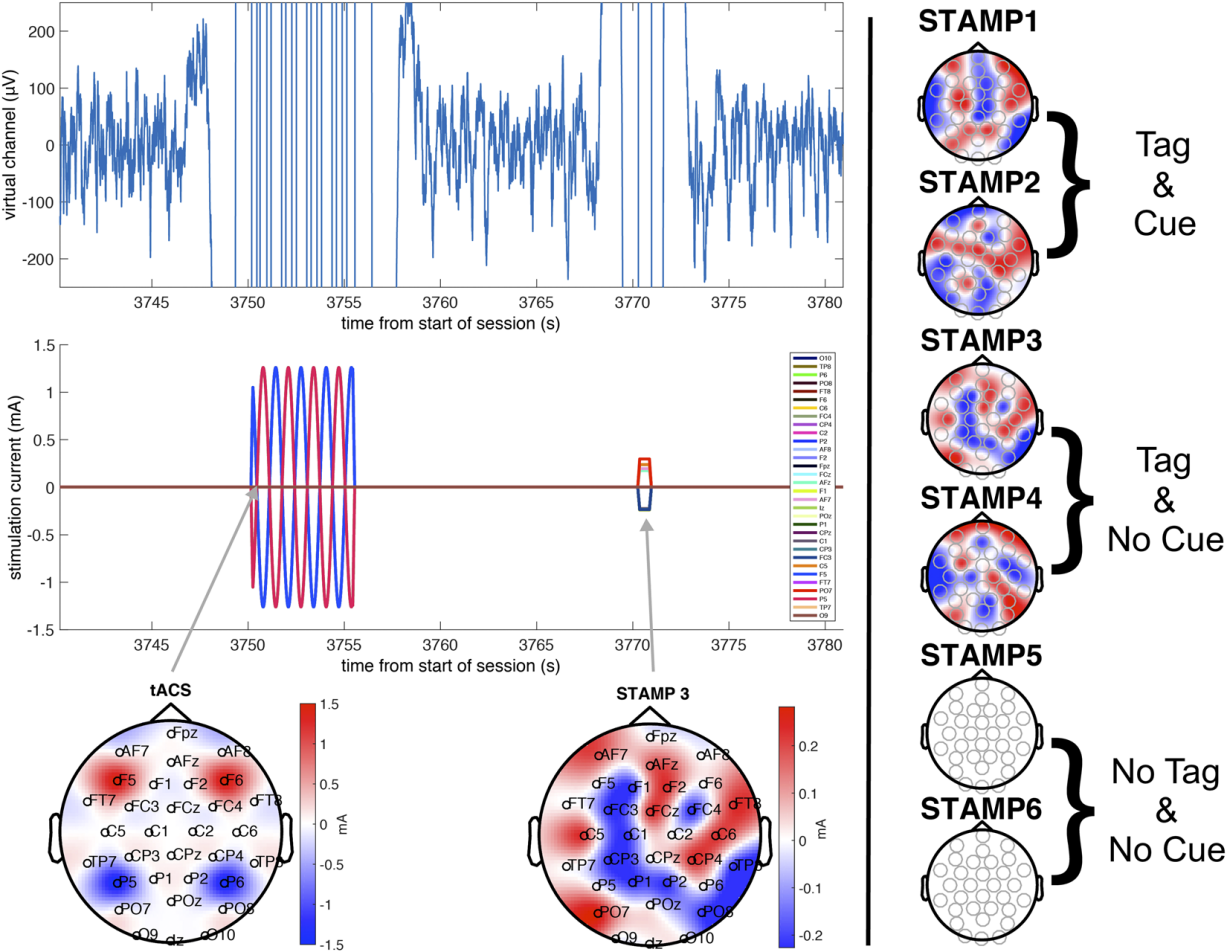
Illustration of the stimulations used in the experiment. Left panel (top) shows time-locked plots of the EEG virtual channel recording used in the SW detection algorithm, filtered from 0.5 to 50 Hz, before, during and after representative tACS and STAMP stimulations; Left panel (middle and bottom) shows stimulation amplitude and matching scalp topography for a representative subject. Here, stimulation is triggered by increases in SW power and is coupled to oscillations in the SW band (0.5 to 1.2 Hz). Clear stimulation artifacts can be seen surrounding the application of both tACS and STAMPs, as well as the return to normal range of amplitudes following the offset. Right panel shows the set of STAMPs assigned to this representative subject, with STAMPs 1 and 2 assigned to the ‘Tag’ condition, i.e., stimulated only during training; STAMPs 3 and 4 assigned to the ‘Tag & Cue’ condition, i.e., stimulated during both training and sleep; and STAMPs 5 and 6 assigned to the Control condition where no currents are applied. Note that the assignment of STAMPS to sequences was randomized for each subject and counterbalanced across subjects.

Online tDCS was delivered during the task using 4 unique spatial patterns of DC currents (namely, STAMPs). Each STAMP is defined as an array of currents across 32 stimulation electrodes located in the 64-channel 10/10 layout (see Table S2 for the particular patterns used). These arrays are optimized through gradient descent to maximize the distance between the intracranial electric fields (cortical orthogonal components) generated by any pair of STAMPs, with the following constraints: the sum of current across the electrodes for each STAMP is zero; for any given STAMP the total injected current does not exceed 2.5 mA; any single electrode must not exceed 1.5 mA; and at least 18 out of the 32 electrodes have a minimum current of 0.15 mA. An original library of 256 STAMPs was created, and a set of 4 STAMPs were randomly selected for use in this experiment. For each subject, these 4 STAMPs were uniquely paired, in random, with four of the target sequences that were assigned to the ‘Tag & Cue’ and ‘Tag & No Cue’ stimulation conditions, and this pairing remained consistent throughout the experiment for that subject. During the training sessions, whenever a target sequence from the Tag & Cue or Tag & No Cue groups initiated, the corresponding STAMP stimulation was delivered throughout the full 8–10 s duration of the sequence with ramp up and ramp down times of 100 ms. The two STAMPs (out of the 4) that were used to stimulate the two sequences belonging to the ‘Tag & Cue’ condition were then re-applied during sleep, as mentioned above. The other two belonging to the ‘Tag’ condition were only applied during training. See Fig. 5 for the scalp topography of the 4 STAMPs used in the experiment (the figure shows a specific representative assignment of those STAMPs to sequences in the ‘Tag & Cue’ and ‘Tag & No Cue’ conditions, though the particular assignment was randomly chosen for each subject, as noted above). Note that the same 4 STAMPs were randomly assigned to these stimulation conditions for each subject in the Active group.

#### Closed-loop stimulation during slow-wave oscillations

Our transcranial current stimulation algorithm was triggered by SW oscillations through the whole night to transiently apply tACS and STAMPs in a closed loop with the oscillations. Based on previous closed-loop phase prediction approaches for augmenting SW oscillations with auditory stimulation^51–53^, our algorithm first detects the presence of SW oscillations, which consist of low frequency synchronized upward and downward deflections of EEG. The algorithm next attempts to match the stimulation frequency and phase with ongoing SW activity such that the maximal stimulation occurs at the up states (positive half waves) of the endogenous SW oscillations. For robust SW oscillation detection, a virtual channel is computed by averaging 13 fronto-parieto-central EEG channels (Cz, FC1, FC2, CP1, CP2, Fz, C4, Pz, C3, F3, F4, P3, P4 in the 10/10 system) to determine the overall synchronous activity of EEG recorded during sleep. The virtual channel allows the observation of moments of relatively high SW power, referred to as ‘SW events’, while averaging out activity of lesser magnitudes on individual channels unrelated to the pattern of SW oscillations. The included channels are stored in a running 5 s buffer. They undergo moving average subtraction with a 1 s window (to mean center the signals at 0 *μV*), and noisy channels exceeding 500 *μV* min-to-max amplitude across the 5 s are rejected before the virtual channel is computed. The buffer is updated with each discrete data fetch operation that gets the new latest data till the point of data request. By the time the buffer is updated, there is a random transmission delay, which needs to be accounted for to plan and precisely time the stimulation intervention in the near future.

The virtual channel data in the buffer is further processed to actually detect the presence of SW oscillations and possibly predict the upcoming up state. The algorithm applies a Fast Fourier Transform (FFT) to these buffered data to determine the power spectrum. Stimulation is planned when the ratio of the cumulative power in the SW band (0.5–1.2 Hz) is more than 20% of the total cumulative power from 0.1 to 250 Hz. If this SW relative power threshold of 0.2 (or 20%) is crossed, the algorithm then filters the data in the SW band with a second-order zero-lag Butterworth filter. Next, a sine wave is fit to the filtered virtual channel using the identified dominant frequency in the SW band, and with the amplitude, offset, and phase parameter values optimized. The sine wave is then projected into the future, identifying the temporal targets that would synchronize tACS or STAMPs to the predicted endogenous SW oscillations. Throughout this process, the dynamic latency associated with data processing is timed using the system clock. Together with distributions of calibrated latencies for data fetch and stimulation commands (mean = 5 ms, SD = 2 ms), which were measured offline, the algorithm estimates the correct time-point to communicate with the hardware to initiate the stimulation. As an example, suppose that at a given moment the algorithm initiates data fetch to populate the buffer with the last five seconds of EEG data. The data then becomes available for processing a few ms (say, 6 ms) into the future based on sampling from the distribution for data fetch latency. Assume it then takes 100 ms for data processing to predict the next up state, which happens to be 600 ms into the future from the starting time-point. If it takes a few ms (say, 7 ms) to physically initiate stimulation based on sampling from the distribution for stimulation command latency, the algorithm would wait 487 ms (600 ms – 100 ms – 7 ms – 6 ms) after the EEG processing step to send the stimulation command to the device.

Based on this SW detection algorithm, two types of non-invasive electrical stimulation were administered in an alternating manner through the sleep period: tACS, matching the frequency and phase of the endogenous SW oscillation to boost their power and induce a longer train; and tDCS, using the STAMP method described earlier, to boost the probability of specific memory replays. tACS was applied in a closed-loop manner because of the mixed behavioral results from various studies (e.g., 7 vs. 31–33) attempting to replicate the original open-loop offset oscillatory protocol of ref.^6^. In addition, given that the intracranial electric fields induced by safe levels of transcranial current stimulation (after shunting and attenuation of scalp-applied currents by skin and skull) are relatively weaker compared to invasive stimulation^33,34^, closed-loop tACS takes advantage of the endogenous oscillations to increase the efficacy of the otherwise difficult task of stimulating from the scalp. That is, instead of inducing SW oscillations *de novo*, closed-loop tACS attempts to prolong already instantiated brain states^16^. STAMPs, on the other hand, were applied in a closed-loop to match the predicted up states of SW oscillations because memory replays in the cortex are associated with those states^38^.

At the start of a given intervention session, tACS was applied (in-phase F5 and F6 in the 10/10 system, and out-of-phase P5 and P6 at 1.5 mA/hemisphere) to the first detected SW event, with stimulation lasting for 5 cycles (cycle being the progression from 0° phase to 360° phase) at the dominant SW frequency of the virtual channel in the 5-s buffer. In some cases, due to hardware and processing delays or targeted up states very close in time to the EEG buffer, the targeted start of the up state stimulation was not possible. In these cases, the algorithm compared the current time to the (now deprecated) stimulation start time, and checked if at least 300 ms of up state stimulation was still possible. If so, the stimulation was initiated immediately and continued throughout the remainder of the predicted up state. In the event that at least 300 ms of up state stimulation was not possible, the algorithm started stimulation at the next upcoming up state based on the continued sine wave projections from the buffer, and was continued until 4 full cycles were completed. Once tACS delivery was completed (i.e., after stimulation offset), the system idled for 3 s to avoid the collection of stimulation artifacts in the data buffer, then resumed the cycle of data update in the buffer. When the next SW event was detected, tDCS STAMP was administered in a similar phase- and frequency-dependent way, though the duration was only for the predicted up state of the SW oscillation. After the delivery of tDCS STAMP was completed, the system again idled for 3 s to avoid stimulation artifacts, then continued data collection in search of the next SW event, at which point tACS was administered, followed by another tDCS STAMP, and so on in an alternating manner. Both tACS and STAMPs had ramp up and ramp down times of 100 ms.

Thus, the closed-loop stimulation system was able to target specific phases of the ongoing endogenous SW oscillations, while adapting parameters continuously in real time in order to minimize temporal inaccuracies due to hardware or processing delays. For Sham subjects, the same algorithm was applied to mark stimulation times without any stimulation being actually applied. An example of a tACS and STAMP pair triggered by endogenous SW oscillations for a given subject is shown in Fig. 5. Note the two tDCS STAMPs for the Tag & Cue condition were always applied in pairs, while alternating with tACS, with the order randomized across pairs through the sleep period. In other words, for every two applications of tACS there were applications of the two ‘Tag & Cue’ STAMPs in a random order.

#### Post-hoc sleep EEG analysis

For sleep staging, sleep EEG data for electrodes C3, C4, O1, O2, Fp1 and Fp2 were filtered with bandpass from 0.5 to 35 Hz, together with EMG data filtered between 10 and 100 Hz. Each 30-second epoch was visually inspected by an experienced technician and assigned a stage of wake, N1, N2, N3/SWS, REM, or movement according to guidelines by the American Academy for Sleep Medicine^54^. Time in each sleep stage was directly derived by summing up all epochs determined to belong to that sleep stage. Percent of time in a sleep stage out of total sleep time was calculated as the amount of time spent in the stage divided by the total amount of time spent asleep (see summary in Table S1).

Stimulation-related analysis of the sleep EEG data was performed using custom-built scripts implemented in Matlab R2016a (The MathWorks) taking advantage of various Fieldtrip^55^ and EEGLAB^56^ functions. EEG data was extracted from sleep sessions and epoched into pre- and post-stimulation windows, which were in turn triggered by SW oscillations. The same process was carried out for the Sham group by estimating where stimulation events would have occurred given the ongoing SW oscillations, and are synonymously referred to here as ‘SW events’.

We validated the up-state detection algorithm on data from Sham nights to avoid large artifacts produced by stimulation in the data from Active nights. Markers were extracted from the time-points of up states predicted by the sine wave fit. Epochs time-locked to the start of predicted up states (−5 to +5 s) were extracted from the EEG virtual channel data and filtered with a 0.5–1.2 Hz bandpass filter. Phase values at the time-point of each start marker were calculated using the Hilbert transform. Mean phase values across trials were calculated for each subject and then submitted to a v-test to examine whether the average across subjects is different from 0 degrees^57^. As stated above for Active nights, in some instances a timed stimulation either did not initiate at the start of an up state or was moved to the next up state, which may have increased the variability of SW phases at the start of stimulation across the night to some degree.

For analysis of the brain response following stimulation, pre-stimulation epochs captured −6.4 s to 0 s before stimulation onset, and post-stimulation epochs captured 0 s to 12.8 s relative to stimulation offset. Here, the temporal duration of the targeted stimulation is 5 cycles at the event-specific dominant frequency within the range of 0.5 to 1.2 Hz (i.e., a range from 4.16 to 10 s) for tACS, and half a cycle for tDCS STAMPs with a range from 0.416 to 1 s. A segment-level artifact removal was done within each epoch by searching in 200 ms sliding windows for a peak to peak voltage change of 500 *μV* within each channel. Any segment that crossed this threshold was marked as bad and interpolated using non-artifact afflicted time-points before and after the marked segments. Any channel that had more than 25% of its segments within a given epoch marked as bad was discarded, and the full epoch for that channel was interpolated using neighboring channels. Any SW event that had more than 80% of its channels exceed the 25% segment threshold was discarded entirely. After segment-level artifact removal, a pass of trial-level removal was done such that any channel that exceeded the peak-to-peak voltage change threshold of 500 *μV* within a given SW event was reconstructed by interpolation of its neighbors, and any SW event in which more than 80% of the channels exceeded that threshold was discarded entirely. Trial sub-selection was done with the constraint that each trial had at least 3 cycles at 4 Hz (approximately 1 s) of usable data both pre and post-stimulation event. Following artifact removal all epochs were truncated to −6.4 to −1 s pre-stimulation and 3 to 12.8 s post-stimulation to ensure no stimulation artifacts lingered in the data. Finally, all epochs were mean centered, bandpass filtered between 0.1 and 125 Hz, bandstop filtered between 59 and 61 Hz, and all channels were re-referenced to the global average across channels.

Time frequency decomposition was done in FieldTrip using Morlet wavelets. Before decomposition, symmetric (mirror) padding was used to extend the pre and post-stimulation event time series to avoid edge artifacts in frequency decomposition. The series of wavelets used in the decomposition started with a width of 4 at the center frequency of 0.5 Hz, and subsequent center frequencies were chosen such that each wavelet was one standard deviation in frequency domain from the previous wavelet. Simultaneously, the wavelet width was increased as a function of center frequency to minimize the combined uncertainty in time and frequency domains, with a starting width of 4 and maximum width of 12. This yielded a Time Frequency Representation (TFR) with 52 approximately log spaced frequency bins from 0.5 to 100 Hz, and equally spaced time bins of 20 ms. For each SW event, spectral power was normalized within each frequency bin by first z-scoring based on a mean and standard deviation estimated over the whole time period (−6.4 to −1 s before the SW event, and 3 to 12.8 s after the SW event). Relative power within each frequency bin was then calculated using a baseline period across SW events by concatenating −3.5 to −3 s from all pre-stimulation periods and estimating a mean and standard deviation from this concatenated time series. These values were then used to z-score within frequency bins both the pre and post periods for each stimulation, to avoid single trial bias in spectral normalization^58^. This z-scored change in power was then averaged across epochs within the Active and Sham groups separately to yield a single channel × time × frequency matrix for each group and subject.

Significant changes in relative power and correlations with behavior were estimated using FieldTrip’s permutation-based clustering algorithm^59^. Spectral power within the range of 0.5 to 4.0 Hz for SW and delta, 4.0 to 8.0 Hz for theta, 8.0 to 12.0 Hz for slow spindles, 12 to 16 Hz for fast spindles, and 17 to 30 Hz for beta were averaged to get an estimate of band-specific power. Using these average power measures, a comparison of the relative change in average power (post-stimulation normalized by pre- stimulation, as described above) for the Active group compared to the relative change in power for the Sham group was made for each channel × time bin between 3 and 10 s from offset of stimulation events. The comparison with Sham was evaluated using an independent samples t-test between the two groups of subjects, and a cluster-based permutation test was performed to determine the significant channel × time bins. Clusters were created by grouping adjacent bins that had an alpha level of *p* < 0.05 (FieldTrip parameters *clusteralpha* and *number of neighbors* were set to 0.05 and 1, respectively). Each cluster was then characterized by the sum of its *t*-values, and a surrogate distribution of clusters, similarly characterized, was created by shuffling the subject labels and repeating the clustering procedure 1000 times. Thus, a clusterwise significance value can be attributed to each observed cluster in reference to its position in the permutation-based surrogate distribution. Here we report any cluster that reached a clusterwise significance less than 0.05 (i.e., 95% of the surrogate clusters had smaller summed *t*-values than the observed cluster) after application of Bonferroni correction for 5 multiple comparisons (for five frequency bands examinations). Any contrast cluster that reached this clusterwise threshold was then used as a mask to perform a subsequent cluster-based permutation test on the correlation between behavior and the significant channel × time bins. In particular, the behavioral measure used for this correlation analysis was the average overnight change in the Distance metric of each subject (for either the Memory items or the Insight items or the average across both, as described in Results section). For both Active and Sham groups separately, correlation coefficients for each significant channel × time bin were calculated within each subject, transformed into a *t*-value, and adjacent bins that had a significance of *p* ≤ 0.05 were clustered together. The same permutation-based significance test was performed as for the contrast clusters, where a surrogate distribution of clusters was created by shuffling the subject labels and repeating the correlation clustering procedure 1000 times. This number of permutations at the alpha level of 0.05 leads to an expected error of ±0.007 in the clusterwise p values. Overall, such hierarchical clustering procedure focuses on extracting correlations that account for performance differences between Active and Sham groups induced by the closed-loop stimulation, rather than correlations that are agnostic to brain stimulation.

## Electronic supplementary material

Video Demonstration of the behavioral task

Supplementary Information

## Data Availability

The datasets generated and/or analyzed during the current study are available from the corresponding authors on request.
