# Supplementary material for: Transcranial Current Stimulation During Sleep Facilitates Insight into Temporal Rules, but does not Consolidate Memories of Individual Sequential Experiences
Source: Sci Rep. 2019 Feb 6;9:1516. doi: 10.1038/s41598-018-36107-7 (PMC6365565; doi:10.1038/s41598-018-36107-7)
Supplement: Supplementary file 2 — Supplementary Information [file 41598_2018_36107_MOESM2_ESM.docx]

Supplementary Information

**Transcranial Current Stimulation During Sleep Facilitates Insight into Temporal Rules, but does not Consolidate Memories of Individual Sequential Experiences.**

Itamar Lerner, Nicholas A. Ketz, Aaron P. Jones, Natalie B. Bryant, Bradley Robert, Steven W. Skorheim, Arno Hartholt, Albert S. Rizzo, Mark A. Gluck, Vincent P. Clark, Praveen K. Pilly

*Corresponding Authors*:

Itamar Lerner Praveen K Pilly

197 University Avenue, Rm 209 HRL Laboratories, LLC

Newark, NJ 07102 Malibu, CA 90265

973-353-3674 323-229-0003

itamar.lerner@gmail.com pkpilly@hrl.com

**This PDF file includes:**

Figs. S1 to S4

Table S1 to S2

Captions for Movie S1

**Other supplementary materials for this manuscript include the following:**

Movie S1

**
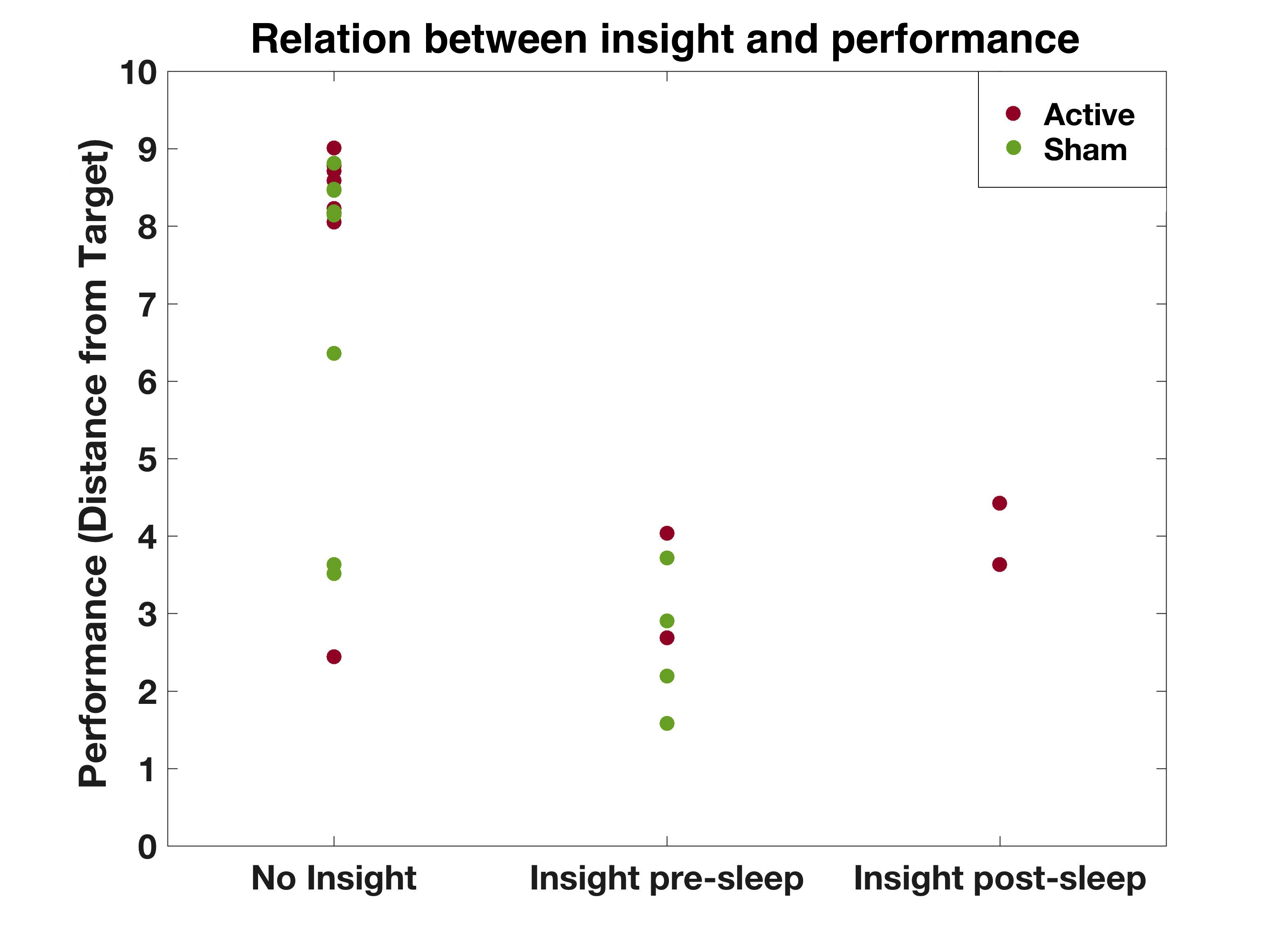
**

**Fig. S1**. Relation between insight learning and task performance. Participants who achieved insight, as determined by post-session strategy questionnaires at the end of training and testing, showed consistently low values in the average Distance metric for items 4 and 5, indicating the ability to predict the location of upcoming targets. Here, the Distance values are computed over the last 5 trials of the relevant session (training session for subjects who either had no insight or gained insight pre-sleep; testing session for subjects who gained insight post-sleep) and averaged over items 4 and 5. Some participants had reduced distance despite having no insight, likely due to memorization of the individual sequences without realizing the hidden rule.


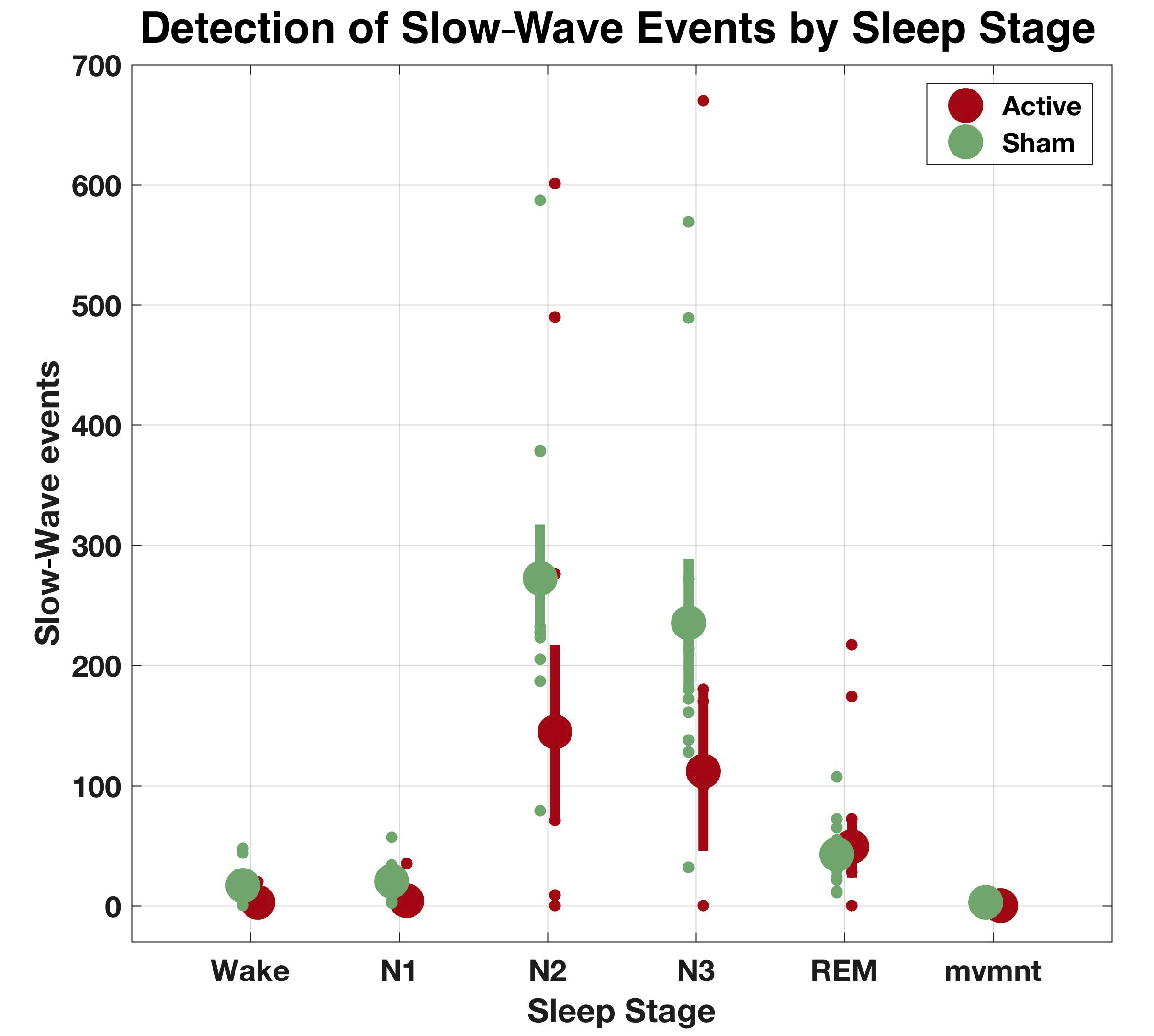


**Fig. S2**. Count of SW events per sleep stage for the Active and Sham groups, showing most events were detected during NREM sleep stages N2 and N3. Large dots and their bars represent the mean and standard errors. Small dots represent individual subjects with available data (some dots are not apparent due to overlap). mvmnt = epochs not classified as one of the sleep stages due to movement.


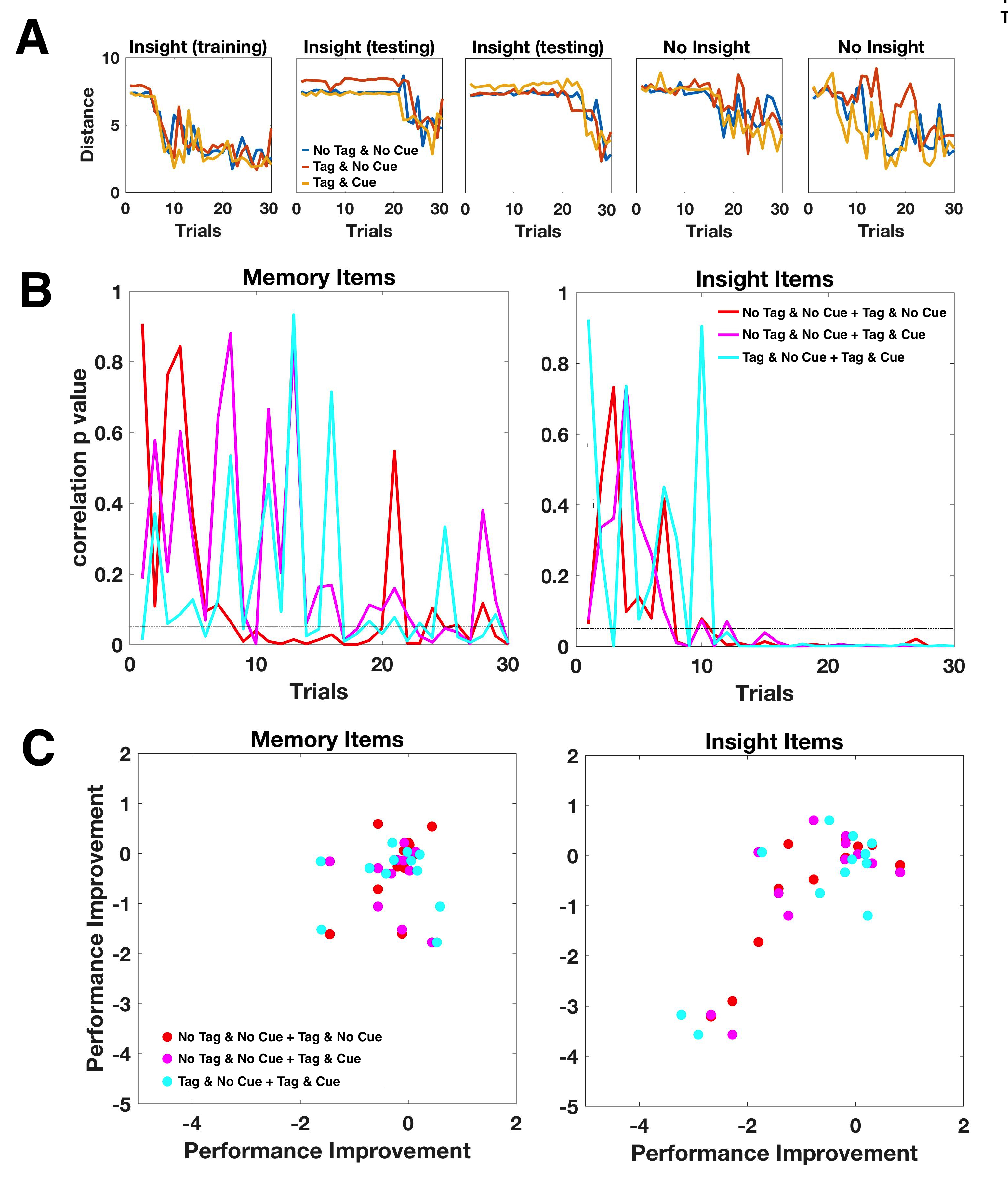


**Fig. S3**. (A) Examples of typical trial-by-trial learning of the Insight items in five different subjects. Trials 1-20 correspond to the training sessions before sleep, and trials 21-30 correspond to the testing session following sleep. Subjects that had insight (either during training or testing) show a sudden improvement in performance for all sequences and thus across all stimulations conditions. Subjects that memorized the individual sequences without having insight show a gradual and/or a less synchronized improvement across the different sequences. (B) *p*-values of the pairwise correlations of performance among the 3 stimulation conditions (‘No Tag & No Cue’ and ‘Tag & No Cue’, ’No Tag & No Cue’ and ‘Tag & Cue’, ‘Tag & No Cue’ and ‘Tag & Cue’) of Active subjects along trials. Dotted line represents the 0.05 significance level. While for Insight items the correlations became highly significant as learning progressed, for Memory items the correlations often did not reach statistical significance. (C) Scatter plots of pairwise comparisons of overnight performance change (computed as the averaged difference between the testing and the last training session) among the 3 stimulation conditions of Active subjects. Overnight performance change in Memory items was uncorrelated whereas that for Insight items was strongly correlated for each of the three comparisons (all *p*’s < 0.008).


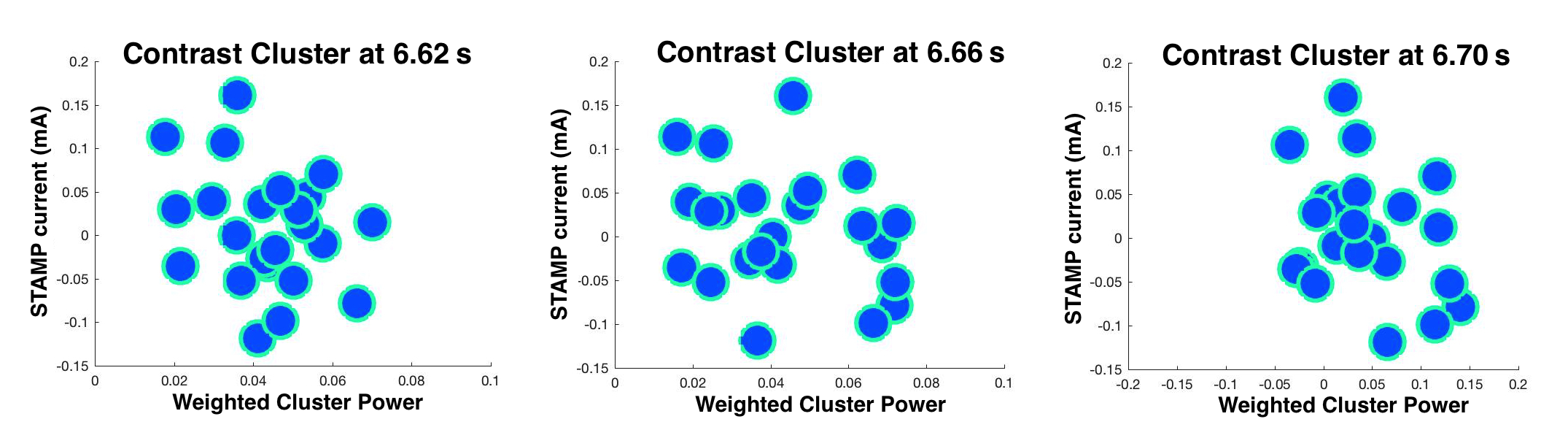


**Fig. S4**. Correlation between the average over STAMPs (detailed in Table S2) and the beta band contrast cluster differentiating Active and Sham subjects (Fig. 4C in the main text). To compute the correlation, we interpolated the currents of each STAMP on a 2D surface (using Matlab’s ‘v4’ spline method) to estimate their influence at EEG channel locations, and averaged over STAMPs.  We then compared this grand averaged pattern of interpolated currents within each channel with the weighted average of the cluster statistic for the corresponding channel, for three different time bins within the cluster (early, at 6.62 s; at the middle of the cluster, at 6.66 s; and late, at 6.70 s. See Fig. 4C).  Here, the weighted average is calculated as the sum of t-values of a given channel within the relevant time bins divided by the total sum of t-values over all channels for those time bins.  Scatter plots indicating these relationships are displayed (each dot represents a different channel). Computing the correlations across channels revealed there were no significant effects (all *p*’s>0.11), indicating that the contrast cluster was not a simple function of the STAMPs. We also computed this correlation using only the 12 channels that had significant t-values within at least one time bin in the cluster (Fig. 4C). Here, the weighted average was calculated as the sum of t-values within significant time bins of a given channel divided by the total sum of t-values in all significant bins of the whole cluster. The correlation with the average STAMP was then computed over those 12 channels alone. This correlation was also not significant (*p* = 0.39).

**Table S1**. Sleep stage statistics.

|  | Time in sleep stage (min) | % time in sleep stage out of Total Sleep Time (TST) |
| --- | --- | --- |
| **Active group** |  |  |
| N1 | 8.17 (5.49) | 1.76 (1.18) |
| N2 | 283.67 (27.49) | 61.06 (6.08) |
| SWS | 98.83 (37.07) | 21.26 (7.94) |
| REM | 74.00 (8.72) | 15.92 (1.84) |
| **Sham group** |  |  |
| N1 | 19.90 (7.72) | 4.28 (1.68) |
| N2 | 269.80 (36.26) | 57.86 (6.19) |
| SWS | 102.30 (23.30) | 22.06 (5.20) |
| REM | 73.65 (28.13) | 15.79 (5.99) |

*Note. Sleep stage statistics for the participants in the experiment. Values represent averages and standard deviations across subjects. Data is based on subjects for whom the full night of data allowed sleep staging. t-tests showed there was no difference between the groups with the exception of sleep stage N1 (p<0.05 for both time in N1 and % time in N1 out of TST), though this effect does not survive multiple comparisons correction.*

**Table S2**: *32-channel tDCS STAMP currents used for tagging 4 of the 6 sequences in the training sessions for the Active group. Values are in units of µA of currents applied. Note the currents for each STAMP sum to zero.*

| Electrode | STAMP #1 | STAMP #2 | STAMP #3 | STAMP #4 |
| --- | --- | --- | --- | --- |
| O10 | 0 | 0 | 0 | 0 |
| TP8 | -323 | 0 | -285 | 0 |
| P6 | 0 | 0 | 0 | 369 |
| PO8 | -188 | 0 | -268 | 246 |
| FT8 | 291 | 299 | 284 | 0 |
| F6 | 0 | 0 | 0 | 0 |
| C6 | 276 | 286 | 284 | -278 |
| FC4 | 0 | 0 | -284 | -276 |
| CP4 | 0 | 301 | 284 | 269 |
| C2 | -284 | 263 | 0 | 282 |
| P2 | 291 | -270 | -279 | -268 |
| AF8 | 302 | 0 | 0 | 297 |
| F2 | -269 | -282 | 284 | 0 |
| Fpz | -188 | -243 | 0 | 242 |
| FCz | -190 | 303 | 284 | 0 |
| AFz | -188 | 253 | 208 | -272 |
| F1 | 0 | 0 | -282 | 0 |
| AF7 | 281 | -283 | 233 | 246 |
| Iz | 0 | -226 | 0 | 0 |
| POz | 0 | -228 | 0 | -274 |
| P1 | 242 | 239 | -278 | 0 |
| CPz | -338 | -179 | 0 | 0 |
| C1 | 286 | 0 | -269 | -281 |
| CP3 | 0 | 0 | -276 | 0 |
| FC3 | 281 | 270 | -279 | 303 |
| C5 | 0 | -315 | 284 | -283 |
| F5 | 0 | 0 | 0 | 0 |
| FT7 | -301 | 286 | 0 | -287 |
| PO7 | 250 | -236 | 355 | 246 |
| P5 | 0 | 0 | 0 | 0 |
| TP7 | -231 | -238 | 0 | -281 |
| O9 | 0 | 0 | 0 | 0 |

**Movie S1.** Demonstration of the behavioral task. Subjects are located in a 3D immersive environment simulating a surveillance task in a Middle Eastern city. Their goal is to aim a crosshair to each character crossing the windows in the building in front of them, and “take a picture” as quickly as possible while the character is still visible.
